# Cross-Cultural Analysis of Consumers’ Avoidance of Snack Food Ingredients Across 13 Countries Using Check-All-That-Apply (CATA) Method

**DOI:** 10.3390/foods14101729

**Published:** 2025-05-13

**Authors:** Yunjeong Cho, Edgar Chambers, Jeehyun Lee

**Affiliations:** 1 Department of Food Science and Nutrition, College of Human Ecology, Pusan National University, Busan 46243, Republic of Korea; yunjeongcho88109@gmail.com; 2 Center for Sensory Analysis and Consumer Behavior, Kansas State University, Manhattan, KS 66502, USA; eciv@ksu.edu

**Keywords:** ingredient, consumer, perception, cross-culture, non-WEIRD, food familiarity, GMO, online survey

## Abstract

Snack foods are increasingly important because of irregular eating patterns in busy lives. Many consumers state that reading ingredients is important to them making rational choices when consuming snacks. This study investigates consumer’s reported avoidance of a wide range of 20 current and potential snack food ingredients. A survey of approximately 630 consumers in each of 13 countries was conducted using a Check-All-That-Apply (CATA) method. Cochran’s Q test was performed to compare percentages of avoidance among countries, and correspondence analysis and cluster analysis were conducted to visualize the similarity of avoidance tendency among countries. Results showed a high tendency to avoid insect powder, SAPP, and BHA, perhaps because of connotations such as disgust or their “non-natural” connotations. The aversion rates for soybean, corn, wheat flour, and pea flour were low. Significant differences (*p* < 0.05) between countries were found for all 20 ingredients. The countries were grouped into seven clusters based on similar avoidance tendencies. This research offers insights into consumer perceptions of snack food ingredients, helping manufacturers understand ingredient avoidance across cultures. These findings support tailored product strategies to enhance food safety policies. Ultimately, the study contributes valuable data for global marketing strategies and promotes innovation in response to health-conscious consumer trends.

## 1. Introduction

Snacks are foods consumed between meals, and their importance in daily diets has increased due to dietary diversification and lifestyle changes [1]. In modern society, students and office workers often simplify or skip meals due to their busy schedules, leading to increased snacking, irregular meal times, and nutritional imbalances. Snacks could help fill nutritional gaps left by main meals and aid in mood enhancement and stress relief [2]. According to a review by Hess et al. [3], in countries like Brazil, Mexico, Canada, the United States, Greece, and France, snacks contribute significantly to daily energy intake. For example, in Brazil, snacks account for 35% of daily energy intake, while in Mexico, they account for 12%; in Canada and the US, they account for 23% and 24%, respectively; in Greece, they account for 33.5%; and in France, they make up 18.5% [4,5,6,7,8,9].

The snack market is highly competitive, with consumers viewing snacks as quick, easy, and relatively inexpensive items. As a result, they tend to rely on experiential attributes such as previous consumption experiences rather than detailed product comparisons. Visual factors like snack form, color, ingredients, and packaging design play an important role in initial purchases. Beyond taste, other factors such as ingredient design also influence snack selection [10]. Consumers primarily check nutrition labels for weight management, health, and nutritional reasons, while those who do not check them often make habitual purchases [11].

Studies on snack selection using cluster analysis include Hartmann et al. [12], who categorized snack consumers into three groups: Cluster 1 with a healthy dietary pattern, mostly older women and educated men; Cluster 2, the largest group, with moderate consumption of sugary drinks and meats; and Cluster 3, predominantly male, with the highest intake of less healthy foods. Lee et al. [13] divided snack demand for preschool children into ‘Family Health Seekers’, ‘Dietary Trend Seekers’ and ‘Eco-seekers’ based on housewives’ lifestyles. Hwang et al. [14] identified health lifestyles in people in their 20s and 30s, classifying them into ‘Appearance Seekers’, ‘Free-living Groups’, ‘Moderate Management Groups’ and ‘Health Disinterest Groups’, with the Free-living Group leading in high-protein snack purchases.

Food neophobia, the reluctance to eat unfamiliar foods, significantly affects food selection, dietary diversity, and nutritional intake [15,16]. It is often influenced by expectations of dislike or unpleasant tastes [17]. A common example is the reluctance to consume edible insects (entomophagy), which has gained attention due to changes in food supply from population growth and urbanization [18]. Although entomophagy is promoted for health, environmental, and economic reasons [19], negative emotions like disgust play a major role in people’s rejection of insect-based foods [18,20].

As health interest grows, the snack market expands, especially with healthy snacks made from organic or local ingredients. Healthy intentions significantly aid in choosing healthier foods [21]. Checking ingredients when buying snacks is crucial for making informed choices; however, research on consumption or purchasing behaviors based on ingredients is limited. This study aims to (1) examine consumer interest in 13 countries related to avoiding snack ingredients and (2) determine if differences in demographic segments influences ingredient avoidance. This should enhance cross-cultural understanding to guide food companies in developing snack products without ingredients that consumers want to avoid.

## 2. Materials and Methods

### 2.1. Participant Profile

Participants were recruited from 13 countries: Australia, Brazil, China, India, Japan, Mexico, Peru, Russia, South Africa, Spain, Thailand, the United Kingdom (UK), and the United States of America (USA). Recruitment was carried out through the online survey company Qualtrics (Provo, UT, USA), with approximately 630 respondents per country, totaling 8191 respondents. These countries were selected to ensure broad representation across continents, encompassing nations with a historical tradition of eating processed foods and those without. Additionally, they reflect a diverse range of dietary habits, from predominantly vegetarian diets to high meat consumption, and varying levels of regulation regarding ingredients. Moreover, the selection also considered the characteristics of the snack food market, including countries with high snack consumption (e.g., the USA, UK and Japan) and emerging markets where snack consumption is rapidly growing (e.g., Brazil, China and India). Consumer behavior and dietary culture differences were also taken into account. Some countries exhibit strong health-conscious trends, while others have notable ingredient aversions. Additionally, traditional food culture varies, with some nations commonly using specific ingredients, while others are experiencing shifts toward more processed snack foods. Given these differences, it was important to compare the perception of general snack food ingredients (e.g., sugar, salt, and corn syrup) with the acceptance of alternative protein sources like insect powder or less familiar ingredients such as Sodium Acid Pyrophophate (SAPP) and Butylated Hydoxyanisole (BHA). Other factors that may influence ingredient avoidance also were considered, such as the potential impact of consumer education levels on ingredient perceptions.

Moreover, only 4 out of the 13 studied countries (Australia, Spain, the UK and the USA) qualify as WEIRD (Western, Educated, Industrialized, Rich, and Democratic), while the remaining 9 are non-WEIRD. This study, therefore, helps to address the limitations of previous research, which has been based predominantly on WEIRD populations. This aligns with longstanding concerns raised by anthropologists: people from WEIRD societies are among the most psychologically atypical groups in the world [22]. The dominance of WEIRD participants in psychological studies poses a significant challenge to understanding human psychology and behavior on a global scale [23]. These considerations help ensure that the selected countries provide a comprehensive and meaningful comparison of global snack food ingredient preferences.

The ratio of ‘man’ to ‘woman’ was evenly allocated at 50%, and the age groups ‘18–34 years’, ‘35–54 years’ and ‘55 years and older’ were also evenly allocated at 33.3%. Additional respondents were included where data was incomplete. Participants did not receive financial incentives for completing the online survey; however, Qualtrics has a reward system in place to compensate for participants’ time and cooperation.

Participants represented 13 countries reflecting cultural, linguistic, traditional, and religious diversity through multicultural international surveys. Egypt was attempted for surveying but was excluded as a target country due to insufficient recruitment of ‘55 years and older’ respondents.

### 2.2. Methodology

The questions for this study were included in a large-scale survey examining various aspects of food beliefs. The total duration of the survey was set between 15 and 20 min to reduce respondent fatigue. The survey was conducted online using the Qualtrics (Provo, UT, USA) platform with consumers recruited by Qualtrics or their partners in each country. Those databases typically contain more than one million consumers per country with some countries having many more consumers in the database. Ethical approval for the involvement of human subjects in this study was granted by the Kansas State University Research Ethics Committee, reference number 8492.

The total questionnaire (including demographic and other questions) was developed in English and then translated into Portuguese (Brazilian), simplified Mandarin, Hindi, Japanese, Spanish (with local adaptation for Mexico, Peru, Spain), Russian, Afrikaans and Thai for use in the appropriate countries (in India and South Africa, each consumer was given the choice to complete the questionnaire in English if preferred). The survey translation process used a variation of the translation, review, adjudication, retesting, and documentation (TRAPD) approach as used by Seninde and Chambers [24] based on Curtarelli and van Houten [25] and Harkness [26]. The English questionnaire was translated by an expert in the subject area who was a native speaker of the language who also spoke/read English. It was then translated back to English by another subject-area-expert native speaker. Then, the two translators worked together (either face to face or online) to check the final translation and ensure the meanings were as intended. A third party was planned to adjudicate any disagreements, but the two translators were able to reach agreement in the few cases where translations did not agree. This procedure has been used for other surveys across multiple languages [20,27,28]. In addition, after initial translation, the Spanish translation was adapted by ‘local’ translators for use by consumers in Mexico, Peru, and Spain because a single “Spanish” word may not be appropriate in the local country dialect. Finally, a “soft launch” in each country with 50 consumers was conducted to test each translated questionnaire to determine if the questionnaires could be successfully understood and completed in the allotted time. Data from those consumers were tracked; no missing data were found, all data were found to be reasonable, and data from screening and validity check questions, which were included to determine if consumers were paying attention, were satisfactory. At that point, questionnaires were approved and used in the test.

Data from one question regarding snack food ingredients that participants would tend to avoid, along with five demographic questions (sex, age, education level, number of adults (18+) in the household, and number of children (17 and lower) in the household), are presented in this paper. A range of potential and current snack food ingredients were assessed using the Check-All-That-Apply (CATA) method (check each ingredient you would avoid if it was listed as an ingredient in a snack food) for 20 snack food ingredients: baking soda, butylated hydroxyanisole (BHA), black beans, canola oil, corn, corn syrup, gluten, insect powder, lecithin, maltodextrins, molasses, pea flour, salt, sodium acid pyrophosphate (SAPP), sodium bicarbonate, sorghum flour, soybeans, sugar, wheat flour, and xanthan gum.

### 2.3. Statistical Analysis

Frequency analysis was performed on the demographic characteristics. Using the CATA data on ingredient avoidance, Cochran’s Q test was conducted to assess whether there were significant differences in CATA responses among the 13 countries. Additionally, correspondence analysis and cluster analysis were performed to group countries with similar characteristics. Because the dataset consists of binomial-type frequency counts, it was necessary to first compute a distance matrix to ensure the stability of clustering results. Thus, PROC DISTANCE in SAS was used to calculate an appropriate dissimilarity matrix, employing the DGOWER distance as the measure of dissimilarity, which is suitable for binary data, before applying PROC CLUSTER. The DGOWER method accepts all measurement levels including ratio, interval, ordinal, nominal, and asymmetric nominal. It standardizes all numeric variables according to their respective types, and the resulting distance values range from 0 to 1 [29]. To investigate differences in CATA responses based on demographic factors, the Mann–Whitney U test was performed by sex, education level, number of adults in the household, and presence of children in the household. The Kruskal–Wallis test was applied to examine differences in CATA responses across different age groups. Data analysis was conducted using XLSTAT^®^ software (version 2024.1.0), SAS^®^ 9.4 statistical software (SAS Institute Inc., Cary, NC, USA), and R (version 4.5.0; The R Foundation for Statistical Computing, Vienna, Austria). Heatmap visualizations were created using the ggplot2 package in RStudio^®^ version 9.4 (Posit Software (version 2024.12.1), PBC, Boston, MA, USA).

## 3. Results and Discussion

### 3.1. Demographics

The demographic characteristics of the participants are presented in Table 1. The total number of participants was 8191, with approximately 630 participants from each of the 13 countries (Australia, Brazil, China, India, Japan, Mexico, Peru, Russia, South Africa, Spain, Thailand, the United Kingdom and the United States). The sex ratio was evenly distributed, with 50% men and 50% women. Age distribution was also evenly allocated, with 33.3% in each of the age groups. In terms of education level, the consumer population was heavily weighted to those with a university degree, with few respondents having only an elementary education. This may reflect the use of or access to electronic media for taking surveys [30]. The number of adults in each household was most commonly two adults (32.2%), with more adults (3+) more common than only single-adult households. More than half of the participants had one or fewer children living in the household, probably as a result of the large number of older (55+) adult households or the emphasis in various countries of population control.

### 3.2. Overall Global Findings

By examining Table 2, the main finding is that there are no snack food ingredients that 100% of people avoid. In fact, there were no snack food ingredients that more than 70% of people avoid. More than 60% of respondents stated they would avoid insect powder, and more than 50% reported they would avoid SAPP and BHA. This may be attributed to the potential disgust, religious restrictions, or low familiarity of these ingredients. It also could be that some ingredients are not perceived as natural [31]. Fewer than 10% of respondents indicated they would avoid soybean, corn, wheat flour and pea flour perhaps because those ingredients are common and considered natural.

As expected, there were some perceptual inconsistencies noted in the data. For example, the reported avoidance of gluten (the main protein in wheat flour) was more than three times that of wheat flour itself, suggesting that the popularity of gluten-free diets in some countries resulted in some consumers focusing on the gluten component more than wheat flour. It also is possible that gluten was considered less “natural” than wheat flour and thus avoided by some people. In addition, many countries are attempting to reduce the use of GMO (genetically modified organisms) foods and many consumers state they avoid GMOs [32]. However, canola oil, soybean, and corn, had low avoidance frequencies (less than 16%) even though these food ingredients are commonly genetically modified (also called bioengineered) (USDA, 2024) [33].

### 3.3. Country Comparisons

The results indicate significant differences among countries (Table 3). Significant differences (*p* < 0.05) were found for all 20 ingredients.

#### 3.3.1. BHA and SAPP

For BHA and SAPP, the frequencies of reported avoidance were significantly higher in Russia (81.1% and 74.8%, respectively) than in other countries. In general, there is growing caution toward food additives in Russia, with a rising preference for natural and organic ingredients due to health concerns [34]. Russian consumers are becoming increasingly aware of food safety and quality, which could influence their perception of synthetic additives like BHA and SAPP. In addition, Russia’s food safety regulations are primarily enforced under the “Federal Service for Surveillance on Consumer Rights Protection and Human Wellbeing” (Rospotrebnadzor) and the “Federal Law on Food Safety”, and the country strictly regulates food additives. In particular, there are stringent controls on specific chemicals and artificial additives that are suspected of being harmful to human health. Because the chemical names of these additives (BHA and SAPP) may give the impression of being less natural and potentially harmful, this likely turns to avoidance by Russian consumers. This is particularly true in the case of SAPP, which a common ingredient in many baking powder formulations, where there is potential that the “chemical”-sounding name alienated consumers. It must be noted that all 13 countries reported a higher avoidance rate of BHA and SAPPs than all other ingredients.

Interestingly, reported avoidance of BHA and SAPP was significantly lower in Japan (34.8% and 31.6%, respectively) than in other countries. That may suggest that many Japanese consumers were more trusting of the safety or necessity of most ingredients than consumers from other countries. Rupprecht [35] found that although the safety of food was important in Japan, it scored lower in importance than in the USA, Germany, China and Thailand. Those authors also found that only 8% of Japanese consumers distrusted label information from producers. Those findings may suggest that Japanese consumers are more likely to trust the importance of manufacturers using various ingredients.

#### 3.3.2. Black Beans

The avoidance of black beans as a snack food ingredient was significantly higher in Thailand (48.4%) than other countries. Notably, the avoidance rate of black beans in Japan is 0.8%, making it the only ingredient among the 20 ingredients across 13 countries with an avoidance rate below 1%. Thai people primarily consume black beans in traditional Thai desserts [36,37]. For example, the most popular black bean desserts are “Khao Lam”, which is sticky rice in bamboo, and “Black Bean in Sweet Coconut Milk”. However, there seem to be no extruded snacks made with black beans in Thailand, which may contribute to the high avoidance rate of black beans as a snack ingredient. On the other hand, in Japan, black beans are regarded as a culturally healthy food. They are particularly used as an ingredient called ‘kuromame’, which is essential in “osechi ryori”, a traditional New Year’s dish consumed to wish for longevity and health [38]. In Japan, legumes are commonly consumed, and black beans, in particular, are used as ingredients in traditional dishes with significant meaning. As a result, the aversion rate is extremely low (0.8%).

#### 3.3.3. Canola Oil

In the case of canola oil, all countries had less than 20% avoidance, while Russia (38.3%) and Spain (39.2%) showed an avoidance rate close to 40%. Because a large part of canola oil is GMO [39], this is thought to reflect the avoidance of GMOs. According to a survey by VTslOM (Russian Public Opinion Research Center), more than half of Russians (59%) believe that consuming GMO products can cause mutations. Additionally, about 83% of Russians support the ban on GMOs, mainly because they perceive GMOs as harmful to human health [40]. Russia also passed strict regulations in 2016, including a ban on GMO imports and cultivation [41]. In addition, Spain is an EU member state, with strict regulations on GMO products [42]. Although some GMO produce is allowed in Spain, most consumers tend to avoid it. There also may be consumers within Spain who do not prefer genetically modified canola oil. There is also a high interest in organic and natural foods in Spain, where canola oil can be avoided as it is considered a likely product to contain GMO ingredients. There may be historical reasons for this, as toxic oil syndrome (TOS), a large-scale food poisoning incident in Spain in 1981, gave a negative perception of canola oil. Of the 20,000 persons affected, approximately 300 died shortly after the onset of the disease and a larger number developed chronic disease [43]. As seen in Table S2, the 55+ age group in Spain shows a significantly higher avoidance of canola oil, which may be due to historical traumatic events. Since this incident, there has been a growing distrust of canola oil in Spain and a tendency to avoid it. In Spain, canola oil is generally not used for human consumption, as olive oil is preferred. Instead, it is mostly used for biodiesel production or as animal feed [44].

#### 3.3.4. Corn

In the case of corn, other countries had an aversion rate of less than 8%, while Thailand had a significantly higher rate of 59.2%. In Thailand, corn is an important agricultural product, and many Thai consumers frequently consume corn due to its health benefits and taste [45]. Additionally, Thailand is a major exporter of high-quality sweet corn [46]. However, Thai consumers tend to enjoy corn on the cob and they may perceive the cob itself as a snack rather than using corn as an ingredient for snack foods. For the Thailand domestic market, Thai consumers are more familiar with the consumption of fresh sweet corn than processed sweet corn [47,48]. These factors may contribute to the high aversion rate of corn as an ingredient for snacks in Thailand.

#### 3.3.5. Gluten vs. Wheat Flour

In the case of gluten, Russia had the highest rate of avoidance at 53.8%, followed by Mexico and India, both with over 40%. While this could be attributed to concerns over celiac disease or non-celiac gluten sensitivity, the relatively lower rate of wheat flour avoidance suggests that the issue may simply be due to the term itself. Wheat flour was perceived as natural by nearly three times as many people in the USA compared to gluten in part because it may be perceived as more highly processed or because of negative dietary connotations in the USA [31]. Familiarity influences consumers’ definition of what is natural. Therefore, it is presumed that gluten is less familiar than wheat flour, leading to a higher avoidance rate for gluten. This appears to be true especially for Russians’ perception of gluten, considering that the prevalence of celiac disease in Russia is less than 1% [49]. Thus, gluten avoidance is unlikely to be from allergy or intolerance issues.

#### 3.3.6. Insect Powder

For insect powder, the frequency of avoidance was significantly lower in China (28.9%), Mexico (44%) and Thailand (44%), whereas other countries generally showed an aversion percentage of approximately 60–70%. This is in line with results found for willingness to eat insect powder in many types of food products in those countries, perhaps because consumers in those countries often eat insect-based or insect-containing foods [50]. Insect powder can improve the texture of foods and increase their taste [51]. Insect powder can also be used as a natural food colorant, imparting a distinctive color to food products [52]. While researchers recognize edible insects as a source of protein and other nutrients, the development of edible insects for use in food products is limited by consumer acceptance because of many people’s natural fear of insects and the disgust associated with insects in food in many countries. Even though traditional insect snacks face challenges in the modern food market, such as changing consumer attitudes and food regulations, they still hold historical and cultural significance. Asia, China, and Thailand have a rich history of consuming insects. As of 2023, the Catalogue of Edible Insect Resources issued by China’s National Health and Health Commission has approved 26 species of insects as new resources. However, insects are currently not included in China’s food catalog lists for its citizens due to various challenges such as market access, dietary concepts, public opinion campaigns, lack of education, and limited large-scale industrial support [51]. Nevertheless, the golden cicada has now become an essential cuisine on the Chinese dining table [53]. Additionally, silkworms were domesticated early in China for silk reeling, and people have a long history of silkworm pupae consumption [54]. In China, wasps have also been widely consumed insects with a long-standing history of inclusion in diets. China also has a long history of consuming locusts [53]. Edible insects are everywhere in Thailand, where Good Agricultural Practice standards were created and widely used for insect farming [55]. For example, fried scorpions and grasshoppers are common street foods in Southeast Asia, while fried grasshoppers are popular foods in Africa, as well as fried scorpions in Thailand and grasshoppers in Mexico [56]. Insect foods have been consumed in certain areas for a long time, but their acceptance in the global market varies tremendously [51]. For example, although the tradition of eating insects exists in Japan, the number of people who eat insects is small, and regulations regarding edible insect ingredients still need to be developed [57]. Gaining consumer recognition and acceptance remains the biggest challenge for insect consumption [58].

#### 3.3.7. Maltodextrins and Xanthan Gum

Maltodextrins, an ingredient with bulking and other properties, and xanthan gum, a stabilizer used in various food products, tended to show the highest levels of avoidance in Russia (75.1% and 61.0%, respectively) and India (49.2% and 47.6%, respectively) compared to other countries. South Africa also showed higher avoidance of maltodextrins (50.2%). This aligns with previous findings for Russia, where there appears to be skepticism toward food additives. In India, skepticism about these ingredients may be higher because, although they are permitted, they are not common in many snack foods, which are often locally made in small shops. In addition, India’s snack market is growing as it becomes westernized, but so far, there is a higher preference for traditional snacks than processed Western snacks and local stores carrying traditional snacks often are used [59,60,61]. Maltodextrin and xanthan gum are commonly used in various food products as food additives. Although they are generally recognized as safe, some consumers perceive them as artificial ingredients, contributing to negative perceptions, particularly among those who seek to avoid synthetic or highly processed components in their diet. This trend may be especially pronounced in some ‘westernized’ countries, such as Australia, the UK, and the USA, where avoidance rates for these ingredients tended to be higher than in some other countries.

#### 3.3.8. Sodium Bicarbonate vs. Baking Soda

In the case of sodium bicarbonate, Thailand had a significantly higher aversion rate than other countries at 55.2%. On the other hand, the aversion rate for baking soda in Thailand was only 26.7%. According to Chambers et al. [31], chemical names are perceived as unnatural by most consumers. The use of a familiar vs. chemical name (baking soda vs. sodium bicarbonate) impacts the perception of naturalness, with the chemical name greatly reducing the perception of being natural. Therefore, sodium bicarbonate may be perceived as less natural compared to baking soda, which likely explains the higher aversion rate towards sodium bicarbonate. It should be noted that in some countries, sodium bicarbonate (or variations such as bicarbonate of soda) is the ‘common’ name used by consumers, which likely is why the two terms did not differ in many countries.

#### 3.3.9. Soybeans

For soybean, the reported avoidance frequency was significantly high in Russia (31.0%), while in other countries it was around 10%. Because a large portion of soybean worldwide is genetically modified, this also seems to reflect Russia’s negative public opinion on GMOs. According to a survey by VTslOM, 54% of Russians stated that they would not purchase products containing GMOs, with particularly strong prejudice against soybeans [62]. On the other hand, Japan had the lowest aversion rate for soybeans at 1.0%. According to Nakamori [63], soybeans are a key component of “Japanese cuisine”, which has been registered as a UNESCO Heritage of Intangible Differentiation and has become an essential part of the Japanese diet. In fact, Japanese people eat many traditional foods made from soybeans, such as shoyu, miso, tofu, natto, etc. [64]. Therefore, these factors may have contributed to Japan’s low soybean avoidance rate.

#### 3.3.10. Sugar

Sugar provides an interesting perspective in this study where the reported avoidance frequency was less than 25% in most countries, but significantly higher in Thailand (35.4%). However, Thailand has a dietary preference for sweetness, using sugar in many traditional dishes and snacks [65,66,67,68]. According to the Thai Health Promotion Foundation (ThaiHealth), Thais consume about 23 teaspoons of sugar per day, which is nearly four times the amount recommended by the World Health Organization [69]. ThaiHealth has warned that excessive sugar intake is associated with heart disease, diabetes, and obesity. In addition, according to Muangsri et al. [70], excessive sugar consumption is one of the important health behaviors that contributes to non-communicable disease (NCD), the leading cause of death in the Thai population. In response to these health concerns, the Thai government introduced a sugar tax in 2017, making it the first in Asia to impose a consumption tax based on the sugar content of beverages to reduce sugar consumption. This policy reportedly led to a 35% increase in the sale of low-sugar alternative beverages and contributed to a decrease in per capita daily sugar consumption from 27 teaspoons in 2017 to 23 teaspoons in 2021. Consumption taxes have been shown to affect perception of products and contribute to lowered consumption [71]. In addition, increased health-conscious consumption may also have contributed to sugar avoidance [67].

We suspect that some of the respondents who reported avoiding sugar in snack foods may also be considering only white, refined, sugar. In many countries, alternative sweeteners such as brown sugar, palm sugar, honey, maple syrup, and others are locally available and may not be considered “sugar”.

#### 3.3.11. Other Ingredients

For corn syrup, the avoidance rate was highest in the USA at 28.9%, showing a significant difference from other countries, though the magnitude of this difference was not large. Corn syrup has been publicly “shamed” in the USA by some organizations who promote healthy lifestyles although other organizations note its common links with other types of sweeteners. Thus, the publicity over corn syrup likely influenced the slightly higher avoidance rates in the USA.

Lecithin and molasses exhibited similar patterns of avoidance across countries, with Mexico, Peru, and Spain exhibiting the highest levels of avoidance (typically over 45%) and Australia, South Africa, UK, USA, and Japan typically having reported avoidance of approximately 20% or less. The higher levels in some countries could be related to their unfamiliarity with the ingredient. In Mexico and Peru, for example, a similar flavor to molasses is derived from other sweetener sources. In some countries, lecithin, which may come from many sources, is often derived from soy and is labeled as “soy lecithin”, which highlights the source of the lecithin. Because we only stated “lecithin”, this may have made the ingredient less familiar.

There were some differences in countries for pea flour, sorghum flour, and salt. Spain had the highest avoidance rates at 12.5% for pea flour and 21.1% for sorghum flour, and Brazil had the highest reported avoidance for salt at 21.3%; the differences among all the countries was small. In fact, those ingredients showed low avoidance levels for all countries.

#### 3.3.12. Overall Comparison

**Figure 1:**
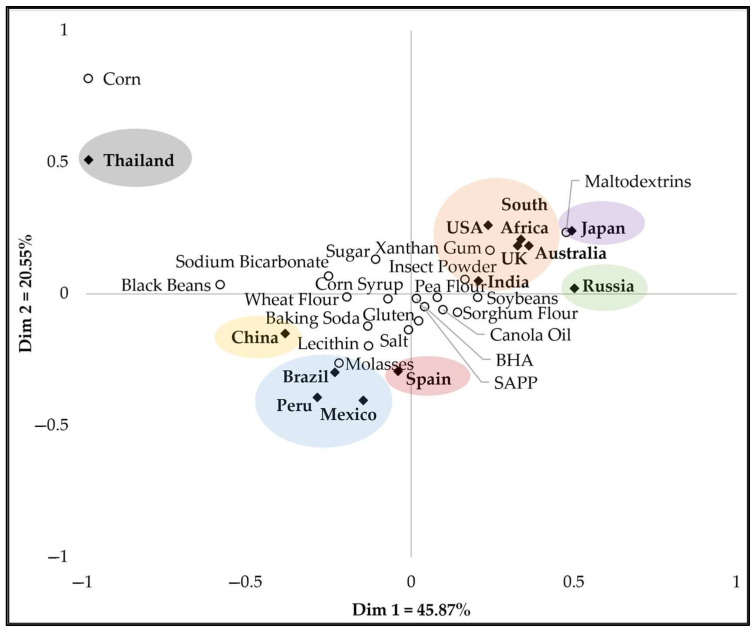
Correspondence analysis biplot of 13 countries’ avoidance of 20 snack food ingredients, selected using CATA. Filled rhombuses (◆) indicate countries; empty circles (○) indicate snack food ingredients. Colored ellipses represent different countries grouped by similarities.

Correspondence analysis allowed visual examination of the distribution of countries and their reported likelihood of avoiding specific ingredients (Figure 1). The results showed that Dimension 1 and Dimension 2 of the correspondence analysis accounted for 45.9% and 20.6% of the variance, respectively, totaling more than two-thirds of the total variation in the data. Australia, India, Japan, Russia, South Africa, the United Kingdom, and the United States were influenced by higher-than-average avoidance of ingredients such as insect powder, maltodextrins, and xanthan gum. The opposite direction was occupied by Brazil, China, Mexico, Peru, Spain, and Thailand, where higher-than-average avoidance of ingredients such as molasses, lecithin, and baking soda was noted. This may be related to familiarity with various ingredients or ingredient names among the countries. It must be noted that corn was positioned near Thailand. The aversion trend for corn, which is around 10% in other countries, shows a significantly higher rate of 60% in Thailand, as noted in Table 3.

Cluster analysis showed seven major clusters, five of which contain only a single country (Figure 1). Cluster 1 includes Australia, India, South Africa, the United Kingdom, and the USA. Clusters 2, 3, and 4 each comprise China, Japan, and Russia in individual clusters, with Japan and China showing some similarities. Cluster 5 includes Brazil, Mexico, and Peru and Clusters 6 and 7 consist of Spain and Thailand, individually. Spain shows some relationship with the countries in Cluster 5. The cluster analysis, which groups countries based on all the data, shows a slightly different relationship among the clusters than the two dimensions in the correspondence analysis.

The findings show that consumers in various countries often have quite different ideas of what ingredients they might avoid in snack foods. Because of the importance of snack foods in the overall diet, this suggests that companies must not only tailor the types and flavors of snack foods for groups of markets but also be diligent about the ingredients they use in various countries in order to ensure that consumers will not avoid various products.

### 3.4. Influence of Demographics on Snack Food Ingredient Avoidance

#### 3.4.1. Sex Influence

**Figure 2:**
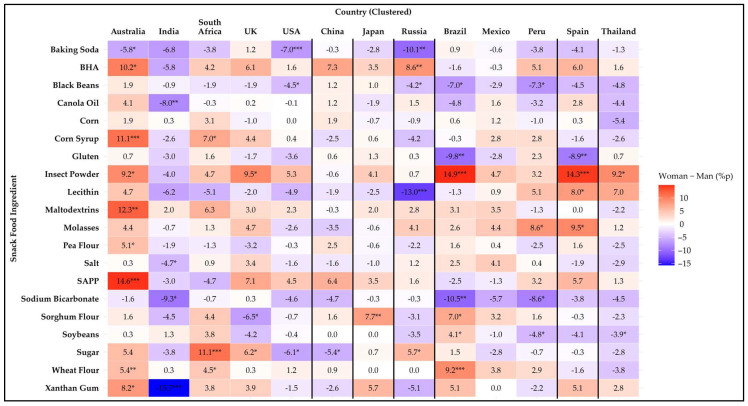
Heatmap of the difference in avoidance rates (%p) for 20 snack food ingredients between women and men across 13 countries. Positive values (red) indicate higher avoidance among women, while negative values (blue) indicate higher avoidance among men. Asterisks denote statistical significance based on Mann–Whitney U test (* *p* ≤ 0.05, ** *p* ≤ 0.01, *** *p* ≤ 0.001).

Figure 2 illustrates sex differences in snack food ingredient avoidance across the countries using a heatmap of percentage point differences. Among the 20 items analyzed, statistically significant differences (*p* < 0.05) were observed for 19 ingredients. Only corn showed no sex differences in avoidance for any country. In addition, 12 of the 13 countries showed sex differences in at least one ingredient. Only Mexico had no significant sex differences for any of the ingredients studied. A detailed summary of avoidance percentages by sex and country is provided in Table S1.

Women were more likely to avoid eight ingredients than men. For BHA, women demonstrated significantly higher avoidance rates in Australia (*p* = 0.011) and Russia (*p* = 0.006). Corn syrup showed significant differences among women and men in Australia (*p* ≤ 0.001) and South Africa (*p* = 0.028), with women displaying higher avoidance rates. Insect powder showed higher avoidance rates for women in Australia (*p* = 0.012), the UK (*p* = 0.013), Brazil (*p* ≤ 0.001), Spain (*p* ≤ 0.001), and Thailand (*p* = 0.020). Maltodextrin, pea flour, and SAPP were avoided more by women only in Australia (*p* = 0.002, *p* = 0.012 and *p* ≤ 0.001, respectively). Molasses was avoided more by women in Peru (*p* = 0.031) and Spain (*p* = 0.017). Wheat flour was avoided more by women in Australia (*p* = 0.006), South Africa (*p* = 0.043), and Brazil (*p* ≤ 0.001).

Men showed higher avoidance than women for six ingredients in at least one country. In the case of baking soda, men exhibited significantly higher avoidance rates than women in Australia (*p* = 0.013), the USA (*p* ≤ 0.001), and Russia (*p* = 0.002). For black beans, men showed significantly higher avoidance rates in the USA (*p* = 0.047), Russia (*p* = 0.032), Brazil (*p* = 0.048), and Peru (*p* = 0.042). Canola oil exhibited significant sex differences (*p* = 0.008) only in India, where men showed a higher tendency to avoid it. Gluten avoidance was significantly higher among men in Brazil (*p* = 0.009) and Spain (*p* = 0.002). Only India showed a significant sex difference (*p* = 0.039) for salt, with men avoiding it more. Sodium bicarbonate was avoided more by men in India (*p* = 0.015), Brazil (*p* = 0.004), and Peru (*p* = 0.020).

Mixed results were found for five ingredients. For lecithin, men in Russia were more likely to avoid it (*p* ≤ 0.001), whereas women in Spain showed higher avoidance rates (*p* = 0.046). Sorghum flour was more likely to be avoided by men in the UK (*p* = 0.012) but by women in Japan (*p* = 0.008) and Brazil (*p* = 0.017). For soybean, women in Brazil showed higher avoidance rates (*p* = 0.040), while men in Peru avoided it more (*p* = 0.050). Sugar was more likely to be avoided by women in South Africa (*p* ≤ 0.001), the UK (*p* = 0.045), and Russia (*p* = 0.015) with men more likely to avoid sugar in the USA (*p* = 0.043) and China (*p* = 0.041). Lastly, xanthan gum also showed mixed results, with women more likely to avoid it in Australia (*p* = 0.028), but in India, men avoided it more (*p* < 0.0001).

Among countries, Australia showed the most sex differences in avoidance of certain ingredients (n = 9), with Brazil in second place (n = 7), and Russia in third (n = 5), and all other countries had four or fewer ingredients that exhibited sex differences.

These findings suggest that sex-specific attitudes toward certain snack ingredients are likely influenced by various factors, including cultural, health-related, or sensory considerations that vary among sexes and countries.

#### 3.4.2. Age Group Comparison

**Figure 3:**
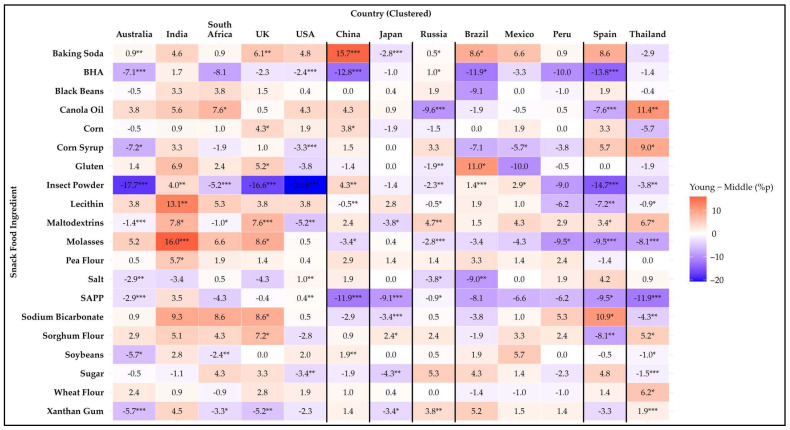
Heatmap of the difference in avoidance rates (%p) for 20 snack food ingredients between younger (18–34 years) and middle-aged (35–54 years) participants across 13 countries. Positive values (red) indicate higher avoidance among younger participants, while negative values (blue) indicate higher avoidance among middle-aged participants. Asterisks denote statistical significance based on Kruskal–Wallis test across the three age groups (* *p* ≤ 0.05, ** *p* ≤ 0.01, *** *p* ≤ 0.001).

**Figure 4:**
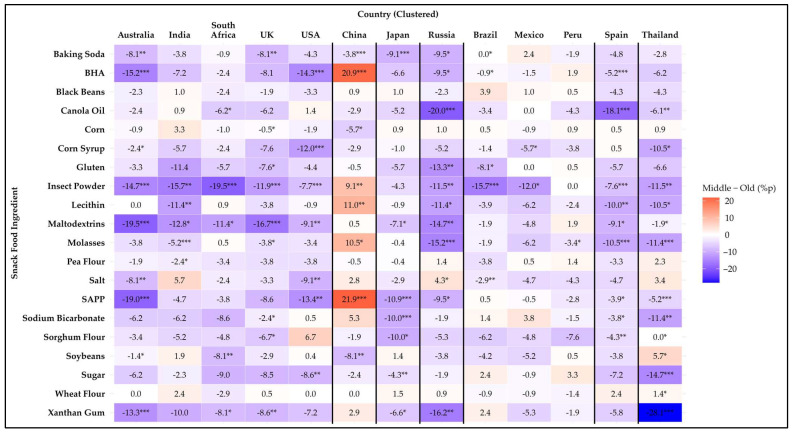
Heatmap of the difference in avoidance rates (%p) for 20 snack food ingredients between middle-aged (35–54 years) and older (55+ years) participants across 13 countries. Positive values (red) indicate higher avoidance among middle-aged participants, while negative values (blue) indicate higher avoidance among older participants. Asterisks denote statistical significance based on Kruskal–Wallis test across the three age groups (* *p* ≤ 0.05, ** *p* ≤ 0.01, *** *p* ≤ 0.001).

**Figure 5:**
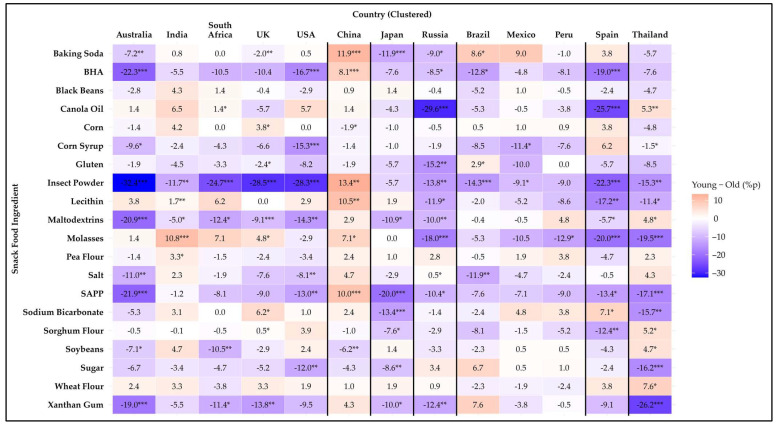
Heatmap of the difference in avoidance rates (%p) for 20 snack food ingredients between younger (18–34 years) and older (55+ years) participants across 13 countries. Positive values (red) indicate higher avoidance among younger participants, while negative values (blue) indicate higher avoidance among older participants. Asterisks denote statistical significance based on Kruskal–Wallis test across the three age groups (* *p* ≤ 0.05, ** *p* ≤ 0.01, *** *p* ≤ 0.001).

Figure 3, Figure 4 and Figure 5 illustrate age differences in snack food ingredient avoidance across the countries using heatmaps of percentage point differences. Among the 20 items analyzed, statistically significant differences (*p* < 0.05) were observed for 19 ingredients. Only black beans showed no age differences in avoidance for any country. Every country had at least one ingredient that showed a difference among age groups. In general, there were more significant differences within countries for age-related differences than those for the sex differences discussed previously. A detailed summary of avoidance percentages by age group and country is provided in Table S2.

The 18–34-year age group showed the highest aversion to two ingredients compared to the groups aged 35–54 and 55 years and older. Pea flour showed a significant difference (*p* = 0.049) only in India, where the 18–34-year group exhibited significantly higher aversion compared to the 35–54-year group. Wheat flour showed a significant difference (*p* = 0.048) only in Thailand, where the 18–34-year group exhibited significantly higher aversion compared to the group aged 55 years and older.

The group aged 55 years and older showed the highest aversion to three ingredients compared to the 18–34- and 35–54-year groups. For corn syrup, the aversion was significantly higher in Australia (*p* = 0.035) and Mexico (*p* = 0.019) compared to the 18–34-year group, significantly (*p* ≤ 0.001) higher in the USA compared to the other groups, and significantly (*p* = 0.032) higher in Thailand compared to the 35–54-year group. For sugar, the aversion was significantly higher in the USA (*p* = 0.004) and Thailand (*p* ≤ 0.001) compared to the other groups, and significantly (*p* = 0.002) higher in Japan compared to the 18–34-year group. For xanthan gum, the aversion was significantly higher in Australia (*p* ≤ 0.001), Russia (*p* = 0.002), and Thailand (*p* < 0.0001) compared to the other groups, and significantly higher in South Africa (*p* = 0.047), the UK (*p* = 0.010), and Japan (*p* = 0.040) compared to the 18–34-year group.

Mixed results were found for fourteen ingredients. For baking soda, the group aged 55 years and older showed the highest aversion in Australia, the UK, Japan, and Russia. In contrast, in China and Brazil, the 18–34-year group demonstrated significantly (*p* < 0.05) higher aversion compared to both of the other groups. For BHA, the group aged 55 years and older showed the highest aversion in Australia, the USA, Russia, Brazil, and Spain. In contrast, in China, the 35–54-year group demonstrated significantly (*p* < 0.0001) higher aversion compared to the other two groups. For canola oil, the highest aversion was among the 18–34-year group in South Africa (*p* = 0.017) and Thailand (*p* = 0.007). In Russia and Spain, the highest aversion was observed in the group aged 55 years and older. For corn, there was a significantly (*p* = 0.048) higher avoidance rate in the 18–34-year group compared to the other two groups in the UK. In China, the group aged 55 years and older had a significantly (*p* = 0.044) higher avoidance rate than the 35–54-year group. For gluten, the highest avoidance rate was in the group aged 55 years and older in both the UK and Russia. In Brazil, the 18–34-year group had the highest avoidance rate, with a significant difference (*p* = 0.048) compared to the 35–54-year group. Insect powder showed age-related differences across eleven countries, except for Japan and Peru. The group aged 55 years and older had the highest avoidance rate in Australia, India, South Africa, the UK, the USA, Russia, Brazil, Mexico, Spain, and Thailand. In contrast, in China, the 18–34-year group had the highest avoidance rate, with a significant difference (*p* = 0.009) compared to the group aged 55 years and older. For lecithin, the 18–34-year group had the highest avoidance rate in India. In China, the 35–54-year group had the highest avoidance rate, and in Russia, Spain, and Thailand, the group aged 55 years and older had the highest avoidance rate. For maltodextrins, the group aged 55 years and older had the highest avoidance rate in Australia, India, South Africa, the UK, the USA, Japan, Russia, and Spain. In contrast, in Thailand, the 18–34-year group had the highest avoidance rate, with a significant difference (*p* = 0.036) compared to the 35–54-year group. For molasses, the 18–34-year group had the highest avoidance rate in India and the UK. In China, the 35–54-year group had the highest avoidance rate. In Russia, Peru, Spain, and Thailand, the 55 years and older group had the highest avoidance rate. For salt, the 55 years and older group had the highest avoidance rate in Australia, the USA, and Brazil. In contrast, in Russia, the 35–54 years group had the highest avoidance rate. For SAPP, the group aged 55 years and older had the highest avoidance rate in Australia, the USA, Japan, Russia, Spain, and Thailand. In contrast, in China, the 35–54-year group had the highest avoidance rate, with significant differences (*p* < 0.0001) among all three groups. For sodium bicarbonate, the 18–34-year group had the highest avoidance rate in the UK and Spain. In contrast, in Japan (*p* ≤ 0.001) and Thailand (*p* = 0.004), the group aged 55 years and older had the highest avoidance rate. For sorghum flour, the 18–34-year group had the highest avoidance rate in the UK and Thailand. In contrast, in Japan and Spain, the group aged 55 years and older had the highest avoidance rate. For soybean, the group aged 55 years and older had the highest avoidance rate in Australia, South Africa, and China. In contrast, in Thailand, the 35–54-year group had the highest avoidance rate, with a significant difference (*p* = 0.037) compared to the group aged 55 years and older.

Among countries, Thailand showed the most age differences in avoidance of certain ingredients (n = 13), with Russia in second place (n = 11), and Australia, the UK, and Spain in third (n = 9 each), and all other countries had eight or fewer ingredients that exhibited age differences. These findings highlight that age-related preferences for snack ingredients vary across countries and may be influenced by cultural, dietary, or sensory factors.

#### 3.4.3. Education Level Influence

**Figure 6:**
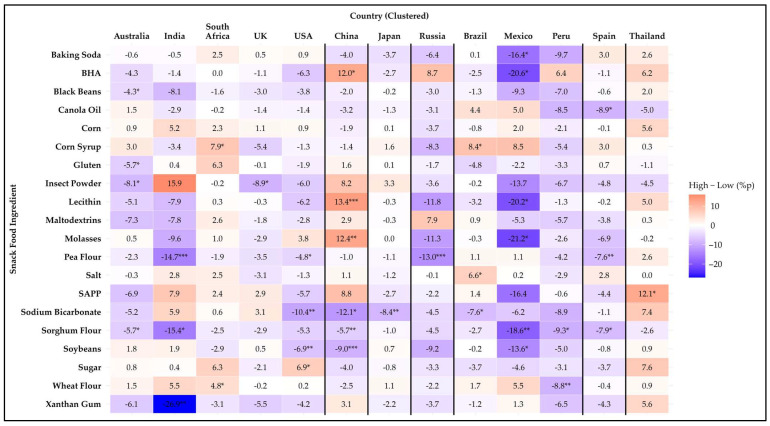
Heatmap of the difference in avoidance rates (%p) for 20 snack food ingredients between higher (college or university graduates) and lower (high school or less) education groups across 13 countries. Positive values (red) indicate higher avoidance among participants with higher education, while negative values (blue) indicate higher avoidance among those with lower education. Asterisks denote statistical significance based on Mann–Whitney U test (* *p* ≤ 0.05, ** *p* ≤ 0.01, *** *p* ≤ 0.001).

Figure 6 illustrates education level differences in snack food ingredient avoidance across the countries using a heatmap of percentage point differences. Among the 20 items analyzed, statistically significant differences (*p* < 0.05) in avoidance depending on education level were observed for 18 ingredients. Only corn and maltodextrin showed no differences across education levels in avoidance for any country. Every country had at least one ingredient that showed a difference among education levels, although the number of differences tended to be smaller than those for age groups. A detailed summary of avoidance percentages by education level and country is provided in Table S3.

Individuals with lower education levels (high school or less) were more likely to avoid ten ingredients than those with higher education levels (college or university graduates). Baking soda avoidance was significantly (*p* = 0.034) higher only in Mexico. Black beans were avoided more only in Australia (*p* = 0.020). Canola oil showed higher avoidance only in Spain (*p* = 0.023). Gluten avoidance was significantly (*p* = 0.029) higher only in Australia. Insect powder showed significant differences in Australia (*p* = 0.029) and the UK (*p* = 0.021), with those in the high school or less group exhibiting higher avoidance rates. Pea flour was more frequently avoided in India (*p* ≤ 0.001), the USA (*p* = 0.040), Russia (*p* < 0.0001), and Spain (*p* = 0.004). Sodium bicarbonate showed higher avoidance among individuals with lower education levels in the USA (*p* = 0.003), China (*p* = 0.011), Japan (*p* = 0.010), and Brazil (*p* = 0.041). Sorghum flour exhibited significant differences in six countries—Australia (*p* = 0.017), India (*p* = 0.012), China (*p* = 0.009), Mexico (*p* = 0.002), Peru (*p* = 0.038), and Spain (*p* = 0.014)—with the high school or less group consistently showing higher avoidance. Soybean was avoided more in the USA (*p* = 0.005), China (*p* ≤ 0.001), and Mexico (*p* = 0.011). Xanthan gum avoidance was significantly (*p* = 0.004) higher only in India.

Individuals with higher education levels (college or university graduates) showed higher avoidance than lower education levels (high school or less) for four ingredients in at least one country. Corn syrup avoidance was more prevalent among the college or university graduate group in South Africa (*p* = 0.017) and Brazil (*p* = 0.018). Salt avoidance was significantly higher in Brazil (*p* = 0.046), with SAPP in Thailand (*p* = 0.029) and sugar in the USA (*p* = 0.024), all among the college or university graduate group.

Mixed results were observed for four ingredients. BHA, lecithin, and molasses showed a similar avoidance pattern: college or university graduates in China exhibited higher avoidance (*p* = 0.012, *p* ≤ 0.001, *p* = 0.003, respectively), while in Mexico, those with a high school education or less reported higher avoidance (*p* = 0.031, *p* = 0.034, *p* = 0.028, respectively). Wheat flour avoidance was higher in the college or university graduates (*p* = 0.034) in South Africa, while the high school or less group showed higher avoidance in Peru (*p* = 0.010).

Among countries, China and Mexico exhibited the most differences in ingredient avoidance based on education level (n = 6 each), followed by Australia and the USA (n = 4 each). Other countries had three or fewer ingredients that showed significant differences across education levels.

These findings suggest that education level influences preferences for specific snack ingredients, likely reflecting varying levels of awareness, access to information, or cultural factors across countries.

#### 3.4.4. Comparison of Number of Adults in Household

**Figure 7:**
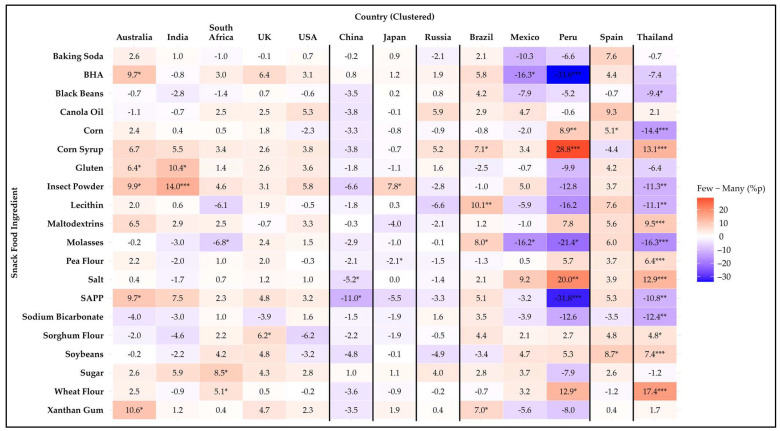
Heatmap of the difference in avoidance rates (%p) for 20 snack food ingredients between participants from households with 1–2 adults and households with 3 or more adults across 13 countries. Positive values (red) indicate higher avoidance among participants from households with 1–2 adults, while negative values (blue) indicate higher avoidance among those from households with 3 or more adults. Asterisks denote statistical significance based on Mann–Whitney U test (* *p* ≤ *0*.05, ** *p* ≤ 0.01, *** *p* ≤ 0.001).

Figure 7 illustrates differences based on the number of adults in the household in snack food ingredient avoidance across the countries using a heatmap of percentage point differences. Among the 20 items analyzed, 18 ingredients showed statistically significant differences (*p* < 0.05). Only baking soda and canola oil showed no significant variation in avoidance for any country. In addition, 11 of the 13 countries showed differences in at least one ingredient. Only the USA and Russia had no significant differences for any of the ingredients studied. A detailed summary of avoidance percentages by the number of adults in the household and country is provided in Table S4.

Households with one to two adults were more likely to avoid eight ingredients than households with two or more adults. For corn syrup, Brazil (*p* = 0.044), Peru (*p* ≤ 0.001), and Thailand (*p* ≤ 0.001) showed significantly higher avoidance rates among the 1–2-adult group. For gluten, the 1–2-adult group showed significantly higher avoidance rates in both Australia (*p* = 0.036) and India (*p* = 0.017). For maltodextrin, only Thailand exhibited a significant difference (*p* < 0.0001), with the 1–2-adult group showing higher avoidance rates. For sorghum flour, the 1–2-adult group reported significantly higher avoidance rates in the UK (*p* = 0.039) and Thailand (*p* = 0.016). For soybean, the 1–2-adult group showed significantly higher avoidance rates in Spain (*p* = 0.028) and Thailand (*p* ≤ 0.001). For sugar, only South Africa showed a significant difference (*p* = 0.013), with the 1–2-adult group reporting higher avoidance rates. For wheat flour, the 1–2-adult group exhibited significantly higher avoidance rates in Peru (*p* = 0.011) and Thailand (*p* < 0.0001). For xanthan gum, the 1–2-adult group showed significantly higher avoidance rates in Australia (*p* = 0.016) and Brazil (*p* = 0.036).

Households with three or more adults were more likely to avoid two ingredients than households with one to two adults. For black beans and sodium bicarbonate, only Thailand showed a significant difference (*p* = 0.031, and *p* = 0.004, respectively), with the group of three or more adults reporting higher avoidance rates.

Mixed results were observed for eight ingredients. For BHA, the 1–2-adult group showed significantly higher avoidance rates in Australia (*p* = 0.037), while the 3+-adult group exhibited higher avoidance rates in Mexico (*p* = 0.045) and Peru (*p* ≤ 0.001). For corn, the 1–2-adults group demonstrated significantly higher avoidance rates in Peru (*p* = 0.010) and Spain (*p* = 0.032), while the 3+-adult group had higher avoidance rates in Thailand (*p* ≤ 0.001). For insect powder, the 1–2-adult group reported significantly higher avoidance rates in Australia (*p* = 0.021), India (*p* ≤ 0.001), and Japan (*p* = 0.043), while the 3+-adult group showed significantly (*p* = 0.009) higher rates in Thailand. For lecithin, the 1–2-adult group had significantly (*p* = 0.009) higher avoidance rates in Brazil, whereas the 3+-adult group showed higher rates in Thailand (*p* = 0.008). For molasses, the 3+-adult group had significantly higher avoidance rates in South Africa (*p* = 0.018), Mexico (*p* = 0.050), Peru (*p* = 0.032), and Thailand (*p* ≤ 0.001), while the 1–2-adult group showed higher rates in Brazil (*p* = 0.043). For pea flour, Japan showed significantly (*p* = 0.035) higher avoidance rates for the 3+-adult group, whereas Thailand showed higher rates for the 1–2-adult group (*p* ≤ 0.001). For salt, the 3+-adult group showed significantly (*p* = 0.030) higher avoidance rates in China, while the 1–2-adult group exhibited higher rates in Peru (*p* = 0.006) and Thailand (*p* < 0.0001). For SAPP, the 1–2-adult group had higher avoidance rates in Australia (*p* = 0.038), while the 3+-adults group showed higher rates in China (*p* = 0.019), Peru (*p* ≤ 0.001), and Thailand (*p* = 0.010).

Among countries, Thailand exhibited the most differences in ingredient avoidance based on the number of adults in the household (n = 13), followed by Peru (n = 7) and Australia (n = 5). Other countries had four or fewer ingredients that showed significant differences across the number of adults in households.

The influence of the number of adults in a household on the avoidance of snack food ingredients highlights the role of household composition in shaping food preferences and consumption behaviors. Households with more adults and those with fewer adults may exhibit different consumption patterns. This suggests that marketers and policymakers should consider household dynamics when addressing dietary choices and promoting healthier snack options.

#### 3.4.5. Comparison of Presence of Children in Households

**Figure 8:**
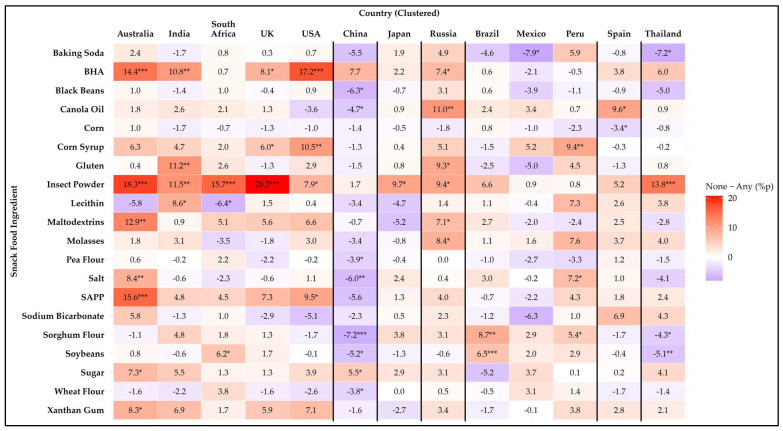
Heatmap of the difference in avoidance rates (%p) for 20 snack food ingredients between participants from households without children and those with children across 13 countries. Positive values (red) indicate higher avoidance among participants from households without children, while negative values (blue) indicate higher avoidance among those with children. Asterisks denote statistical significance based on Mann–Whitney U test (* *p* ≤ 0.05, ** *p* ≤ 0.01, *** *p* ≤ 0.001).

Figure 8 illustrates differences based on the presence of children in the household in snack food ingredient avoidance across the countries using a heatmap of percentage point differences. Among 20 items analyzed, significant differences (*p* < 0.05) were observed for 19. Only sodium bicarbonate showed no differences in avoidance for any country. Every country had at least one ingredient that showed a difference based on the presence of children in households. A detailed summary of avoidance percentages by the presence of children in the household and country is provided in Table S5.

Households without children exhibited significantly higher avoidance rates for nine ingredients. BHA was avoided more in Australia (*p* ≤ 0.001), India (*p* = 0.008), the UK (*p* = 0.047), the USA (*p* < 0.0001), and Russia (*p* = 0.018). Corn syrup avoidance was significantly higher in the UK (*p* = 0.039), the USA (*p* = 0.007), and Peru (*p* = 0.009). Gluten was avoided more in India (*p* = 0.005) and Russia (*p* = 0.020). Insect powder showed the most widespread avoidance differences, with households without children reporting significantly higher avoidance in Australia (*p* < 0.0001), India (*p* = 0.002), South Africa (*p* < 0.0001), the UK (*p* < 0.0001), the USA (*p* = 0.046), Japan (*p* = 0.027), Russia (*p* = 0.013), and Thailand (*p* ≤ 0.001). Maltodextrin avoidance was more pronounced in Australia (*p* = 0.002) and Russia (*p* = 0.038). Molasses was significant (*p* = 0.029) only in Russia, where households without children demonstrated greater avoidance. SAPP was reported higher avoidance in households without children in Australia (*p* ≤ 0.001) and the USA (*p* = 0.025). Sugar avoidance was significantly higher among households without children in Australia (*p* = 0.039) and China (*p* = 0.041). Xanthan gum was significant (*p* = 0.036) only in Australia, with households without children reporting higher avoidance rates.

Households with children showed significantly higher avoidance rates for five ingredients than households without children. Baking soda was avoided more in Mexico (*p* = 0.017) and Thailand (*p* = 0.042). Black beans, pea flour, and wheat flour were avoided more only in China (*p* = 0.012, *p* = 0.021, and *p* = 0.038, respectively). Corn was significant (*p* = 0.018) solely in Spain, with higher avoidance by households with children.

Five ingredients showed mixed avoidance patterns. Canola oil was avoided more by households without children in Russia (*p* = 0.005) and Spain (*p* = 0.015), but households with children avoided it more in China (*p* = 0.011). Lecithin was avoided more by households without children in India (*p* = 0.023) but by households with children in South Africa (*p* = 0.049). Salt was avoided more by households without children in Australia (*p* = 0.004) and Peru (*p* = 0.015) but by households with children in China (*p* = 0.003). For sorghum flour, households without children avoided it more in Brazil (*p* = 0.003) and Peru (*p* = 0.048) while, households with children avoided it more in China (*p* < 0.0001) and Thailand (*p* = 0.019). Soybean also showed mixed results, with households without children reporting higher avoidance in South Africa (*p* = 0.021) and Brazil (*p* ≤ 0.001), while households with children avoided it more in China (*p* = 0.022) and Thailand (*p* = 0.008).

Among countries, China exhibited the most differences in ingredient avoidance based on child presence (n = 8), followed by Australia (n = 7) and Russia (n = 6). Other countries showed four or fewer differences in ingredient avoidance patterns.

These findings suggest that the presence of children in households influences ingredient avoidance behaviors, though patterns vary significantly by ingredient and country. For example, households without children tend to exhibit broader avoidance of processed or less familiar ingredients, such as BHA, maltodextrin, and insect powder. These results highlight the importance of considering household composition and cultural factors when analyzing consumer behavior toward food ingredients. Addressing these differences could help food manufacturers and policymakers develop targeted strategies to meet diverse consumer needs.

### 3.5. Limitations of Study

Several limitations should be noted in this study. The first is that respondents were tested electronically, which means that only consumers who have electronic devices and access to the internet could participate in the study. The databases used are populated with a broad range of consumers but only those with access to the internet are included. Although the number of internet users (those who actually went online within a 3-month period) is high (>70% in 2021) in every country except India, where the rate was 46%, some individuals are not accessible using this method and, therefore, are excluded from this type of survey [30]. This is noted with the higher-than-average education level of the participants across the countries. However, it is important to note that differences in ingredient avoidance between high school or less group and college or university graduate group were smaller than differences observed across other demographic factors, suggesting that education level had a relatively minor impact on ingredient avoidance.

A second limitation of this study is that it focused on whether or not consumers would avoid certain snack food ingredients and did not ask consumers why they would avoid those ingredients. Thus, the survey only provides information about the avoidance rates of these ingredients. Although we speculate on the reasons, future work needs to be conducted to determine the specific reasons behind consumers’ avoidance of snack food ingredients. Such work is necessary to determine whether it is possible to adapt information campaigns to make those ingredients more acceptable to consumers or whether the alternative ingredients need to be used in snack foods.

## 4. Conclusions

This study identified the perceptions of 8191 consumers from 13 countries regarding 20 snack food ingredients. Approximately 630 consumers from each country reported their aversion rates for a range of representative snack food ingredients. The overall consumer aversion rates were highest for insect powder, SAPP, and BHA, each exceeding 50%, while the aversion rates for soybean, corn, wheat flour, and pea flour typically were below 10%. The analysis revealed significant differences between countries for all 20 snack food ingredients, and cluster analysis grouped countries with similar aversion trends into a total of seven clusters. Additionally, the study examined how factors such as sex, age group, education level, the number of adults in the household, and the presence of children in the household influenced the aversion to snack food ingredients. The findings can provide valuable insights for snack food manufacturers, helping them to develop tailored product strategies for each country and contributing to global marketing strategies.

## Figures and Tables

**Table 1:** Demographic information of participants.

Demographic Characteristics	Variables	Frequency (n)	Percentage (%)
Sex	Man	4098	50.0
	Woman	4093	50.0
Age	18–34	2730	33.3
	35–54	2731	33.3
	55+	2730	33.3
Highest Degree earned	Primary school or less	187	2.3
	High School	2010	24.5
	College or University graduate	5994	73.2
Number of Adults 18+ in Household	1	851	10.4
	2	2634	32.2
	3	2225	27.2
	4	1337	16.3
	5	909	11.1
	6	235	2.9
Number of Children <18 in Household	0	4409	53.8
	1	2131	26.0
	2	1229	15.0
	3	314	3.8
	4 or more	108	1.3

**Table 2:** Frequency and percentage of snack food ingredient avoidance among all participants (n = 8191).

Snack Food Ingredients	Frequency (n)	Percentage (%)
Insect Powder	4964	60.6
Sodium Acid Pyrophosphate (SAPP)	4410	53.8
Butylated Hydroxyanisole (BHA)	4402	53.7
Xanthan Gum	2456	30.0
Molasses	2456	30.0
Lecithin	2388	29.2
Maltodextrins	2354	28.7
Sodium Bicarbonate	2120	25.9
Gluten	2100	25.6
Corn Syrup	1596	19.5
Baking Soda	1501	18.3
Sugar	1478	18.0
Canola Oil	1256	15.3
Black Beans	1112	13.6
Sorghum Flour	983	12.0
Salt	934	11.4
Soybeans	808	9.9
Corn	644	7.9
Wheat Flour	589	7.2
Pea Flour	481	5.9

**Table 3:** Significant differences based on Cochran’s Q test in percentage (%) avoidance of ingredients in snack foods for consumers in 13 countries ^1^.

Snack Food Ingredients	Cluster 1					Cluster 2	Cluster 3	Cluster 4	Cluster 5			Cluster 6	Cluster 7	*p*-Value
	Australia	India	South Africa	UK ^2^	USA ^2^	China	Japan	Russia	Brazil	Mexico	Peru	Spain	Thailand	
Baking Soda	9.2 ^f3^	32.9 ^ab^	11.1 ^ef^	7.6 ^f^	7.3 ^f^	17.0 ^de^	11.6 ^ef^	21.0 ^cd^	16.7 ^de^	20.0 ^cd^	37.1 ^a^	20.2 ^cd^	26.7 ^bc^	<0.0001
BHA	50.8 ^cde^	51.9 ^cde^	51.9 ^cde^	41.0 ^fg^	47.0 ^ef^	60.2 ^bc^	34.8 ^g^	81.1 ^a^	48.7 ^ef^	58.9 ^bcd^	63.2 ^b^	59.5 ^bc^	49.7 ^def^	<0.0001
Black Beans	5.4 ^de^	5.6 ^de^	6.3 ^cde^	6.3 ^cde^	8.9 ^cd^	11.1 ^cd^	0.8 ^e^	6.2 ^cde^	26.7 ^b^	12.5 ^c^	28.1 ^b^	10.2 ^cd^	48.4 ^a^	<0.0001
Canola Oil	9.7 ^de^	17.1 ^b^	9.4 ^de^	12.2 ^bcde^	10.2 ^cde^	5.7 ^e^	6.3 ^e^	38.3 ^a^	13.8 ^bcd^	8.4 ^de^	12.1 ^bcde^	39.2 ^a^	16.8 ^bc^	<0.0001
Corn	2.5 ^bc^	4.9 ^bc^	4.4 ^bc^	4.0 ^bc^	7.6 ^b^	6.0 ^bc^	1.0 ^c^	2.1 ^c^	2.2 ^c^	1.9 ^c^	3.0 ^bc^	3.3 ^bc^	59.2 ^a^	<0.0001
Corn Syrup	18.9 ^cde^	16.8 ^de^	19.7 ^bcde^	14.3 ^ef^	28.9 ^a^	8.6 ^fg^	6.0 ^g^	13.8 ^ef^	26.2 ^abc^	22.4 ^abcd^	26.2 ^abc^	24.6 ^abc^	27.0 ^ab^	<0.0001
Gluten	12.1 ^f^	40.3 ^bc^	26.5 ^e^	11.0 ^fg^	17.0 ^f^	3.5 ^g^	9.5 ^fg^	53.8 ^a^	33.5 ^cde^	44.3 ^b^	36.3 ^bcd^	15.2 ^f^	30.2 ^de^	<0.0001
Insect Powder	70.0 ^ab^	68.4 ^ab^	74.8 ^a^	65.6 ^ab^	68.9 ^ab^	28.9 ^d^	65.2 ^b^	66.3 ^ab^	62.4 ^b^	44.0 ^c^	66.0 ^ab^	63.3 ^b^	44.0 ^c^	<0.0001
Lecithin	16.0 ^ef^	32.4 ^d^	21.0 ^e^	14.9 ^ef^	20.2 ^e^	21.0 ^e^	7.9 ^f^	30.3 ^d^	39.0 ^bcd^	41.4 ^bc^	46.3 ^ab^	51.9 ^a^	36.5 ^cd^	<0.0001
Maltodextrins	39.8 ^c^	49.2 ^b^	50.2 ^b^	32.4 ^cd^	40.3 ^c^	3.0 ^f^	27.3 ^d^	75.1 ^a^	8.3 ^ef^	16.3 ^e^	7.9 ^ef^	15.6 ^e^	8.1 ^ef^	<0.0001
Molasses	13.0 ^e^	27.5 ^d^	14.9 ^e^	14.1 ^e^	9.8 ^ef^	24.0 ^d^	3.2 ^f^	37.9 ^c^	41.9 ^c^	51.1 ^ab^	55.1 ^a^	51.7 ^ab^	45.4 ^bc^	<0.0001
Pea Flour	7.0 ^bc^	6.0 ^bcd^	7.0 ^bc^	6.0 ^bcd^	9.0 ^ab^	4.4 ^cd^	1.6 ^d^	4.3 ^cd^	3.3 ^cd^	4.6 ^bcd^	6.0 ^bcd^	12.5 ^a^	4.4 ^cd^	<0.0001
Salt	14.1 ^b^	9.4 ^bcd^	12.9 ^bc^	14.4 ^b^	11.9 ^bc^	6.8 ^cde^	2.4 ^e^	3.5 ^de^	21.3 ^a^	14.4 ^b^	15.4 ^ab^	14.0 ^b^	7.8 ^cde^	<0.0001
SAPP	52.1 ^def^	57.6 ^bcd^	56.0 ^bcd^	41.7 ^g^	45.2 ^efg^	43.5 ^fg^	31.6 ^h^	74.8 ^a^	53.3 ^cde^	59.4 ^bcd^	65.1 ^b^	57.1 ^bcd^	62.5 ^bc^	<0.0001
Sodium Bicarbonate	14.8 ^efg^	35.6 ^b^	22.9 ^cde^	11.3 ^fg^	24.8 ^cd^	38.3 ^b^	18.9 ^def^	8.4 ^g^	30.6 ^bc^	19.0 ^def^	31.3 ^bc^	25.4 ^cd^	55.2 ^a^	<0.0001
Sorghum Flour	9.7 ^cdef^	12.1 ^bcd^	9.2 ^def^	11.7 ^bcde^	15.9 ^abc^	5.2 ^f^	15.6 ^abc^	10.2 ^bcdef^	16.2 ^ab^	10.8 ^bcdef^	12.9 ^bcd^	21.1 ^a^	5.6 ^ef^	<0.0001
Soybeans	9.0 ^bc^	5.1 ^cd^	13.3 ^b^	8.1 ^bc^	10.6 ^bc^	8.6 ^bc^	1.0 ^d^	31.0 ^a^	6.8 ^c^	8.4 ^bc^	10.3 ^bc^	9.7 ^bc^	6.3 ^cd^	<0.0001
Sugar	22.4 ^bc^	17.1 ^bcd^	23.0 ^b^	18.7 ^bcd^	17.6 ^bcd^	12.5 ^def^	6.7 ^f^	9.5 ^ef^	16.3 ^bcde^	17.0 ^bcd^	15.2 ^cde^	23.0 ^b^	35.4 ^a^	<0.0001
Wheat Flour	6.5 ^cde^	5.2 ^cde^	8.3 ^abc^	5.6 ^cde^	7.9 ^bc^	5.6 ^cde^	2.5 ^de^	1.6 ^e^	12.2 ^ab^	12.4 ^ab^	6.8 ^cd^	5.9 ^cde^	13.0 ^a^	<0.0001
Xanthan Gum	33.0 ^cd^	47.6 ^b^	38.7 ^c^	32.5 ^cd^	35.9 ^cd^	7.6 ^g^	21.6 ^ef^	61.0 ^a^	22.9 ^ef^	15.6 ^fg^	15.4 ^fg^	29.8 ^de^	28.1 ^de^	<0.0001

^1^ Cell background colors, white to red, represent low to high levels of avoidance of ingredients in snack foods. Color codes are visualized in ggplot2 in R, version 4.5.0, and RStudio, version 2024.12.1. ^2^ Country abbreviations, UK = United Kingdom; USA = United States of America. ^3,a,b,c,d,e,f,g,h^ letters indicate significant differences between countries of each snack food ingredient. Post hoc pairwise comparison analysis was conducted using the critical difference (Sheskin) procedure.

## Data Availability

The original contributions presented in this study are included in the article/Supplementary Material. Further inquiries can be directed to the corresponding author.

## Supplementary Materials

The supporting information can be downloaded at https://www.mdpi.com/article/10.3390/foods14101729/s1, Table S1: Significant differences based on Mann-Whitney U test in percentage (%) avoidance of ingredients in Snack foods by sex for consumers in 13 countries (data shown as Man (%), Woman (%), and *p*-value); Table S2: Significant differences based on Kruskal-Wallis test in percentage (%) avoidance of ingredients in Snack foods by age for consumers in 13 countries (data shown as 18–34 years (%), 35–54 years (%), 55 years and older (%), and *p*-value); Table S3: Significant differences based on Mann-Whitney U test in percentage (%) avoidance of ingredients in Snack foods by education level for consumers in 13 countries (data shown as High school or less (%), College or university graduates (%), and *p*-value); Table S4: Significant differences based on Mann-Whitney U test in percentage (%) avoidance of ingredients in Snack foods by number of adults in household for consumers in 13 countries (data shown as households with 1–2 adults (%), households with 3 or more adults (%), and *p*-value). Table S5: Significant differences based on Mann-Whitney U test in percentage (%) avoidance of ingredients in Snack foods by presence of children in household for consumers in 13 countries (data shown as households without children (%), households with children (%), and *p*-value).
