# Supplementary material for: Cross-Cultural Analysis of Consumers’ Avoidance of Snack Food Ingredients Across 13 Countries Using Check-All-That-Apply (CATA) Method
Source: Foods. 2025 May 13;14(10):1729. doi: 10.3390/foods14101729 (PMC12111002; doi:10.3390/foods14101729)
Supplement: Supplementary file 1 [file foods-14-01729-s001.zip › foods-3566459-supplementary.pdf]

Table S1. Significant differences based on Mann-Whitney U test in percentage (%) avoidance of ingredients in Snack foods by sex for consumers in 13 countries (data shown as Man (%), Woman (%), and *p*-value).

Snack food ingredients	Data type	Cluster 1					Cluster 2	Cluster 3	Cluster 4	Cluster 5			Cluster 6	Cluster 7
		Australia	India	South Africa	UK[1]	USA[1]	China	Japan	Russia	Brazil	Mexico	Peru	Spain	Thailand
Baking Soda	Man (%)	**12.1**[2]	36.2	13.0	7.0	**10.8**	17.1	13.0	**26.0**	16.2	20.3	39.0	22.2	27.3
	Woman (%)	**6.3**	29.4	9.2	8.2	**3.8**	16.8	10.2	**15.9**	17.1	19.7	35.2	18.1	26.0
	p-value	**0.013**	0.071	0.129	0.564	**≤0.001**	0.916	0.263	**0.002**	0.749	0.842	0.323	0.197	0.719
BHA	Man (%)	**45.7**	54.9	49.8	37.9	46.2	56.5	33.0	**76.8**	49.5	59.0	60.6	56.5	48.9
	Woman (%)	**55.9**	49.1	54.0	44.0	47.8	63.8	36.5	**85.4**	47.9	58.7	65.7	62.5	50.5
	p-value	**0.011**	0.140	0.300	0.121	0.687	0.061	0.358	**0.006**	0.691	0.936	0.187	0.123	0.691
Black Beans	Man (%)	4.4	6.0	7.3	7.3	**11.1**	10.5	0.3	**8.3**	**30.2**	14.0	**31.7**	12.4	50.8
	Woman (%)	6.3	5.1	5.4	5.4	**6.6**	11.7	1.3	**4.1**	**23.2**	11.1	**24.4**	7.9	46.0
	p-value	0.291	0.596	0.328	0.317	**0.047**	0.613	0.179	**0.032**	**0.048**	0.279	**0.042**	0.065	0.232
Canola Oil	Man (%)	7.6	**21.3**	9.5	12.1	10.2	5.1	7.3	37.5	16.2	7.6	13.7	37.8	19.0
	Woman (%)	11.7	**13.3**	9.2	12.3	10.1	6.3	5.4	39.0	11.4	9.2	10.5	40.6	14.6
	p-value	0.080	**0.008**	0.892	0.927	0.979	0.493	0.328	0.682	0.084	0.474	0.222	0.463	0.136
Corn	Man (%)	1.6	4.8	2.9	4.5	7.6	5.1	1.3	2.5	1.9	1.3	3.5	3.2	61.9
	Woman (%)	3.5	5.1	6.0	3.5	7.6	7.0	0.6	1.6	2.5	2.5	2.5	3.5	56.5
	p-value	0.129	0.862	0.053	0.530	0.982	0.316	0.413	0.401	0.590	0.244	0.485	0.825	0.169
Corn Syrup	Man (%)	**13.3**	18.1	**16.2**	12.1	28.7	9.8	5.7	15.9	26.3	21.0	24.8	25.4	28.3
	Woman (%)	**24.4**	15.5	**23.2**	16.5	29.1	7.3	6.3	11.7	26.0	23.8	27.6	23.8	25.7
	p-value	**≤0.001**	0.385	**0.028**	0.119	0.901	0.255	0.738	0.134	0.928	0.390	0.415	0.644	0.473
Gluten	Man (%)	11.7	41.9	25.7	11.8	18.8	3.2	8.9	53.7	**38.4**	45.7	35.2	**19.7**	29.8
	Woman (%)	12.4	38.9	27.3	10.1	15.2	3.8	10.2	54.0	**28.6**	42.9	37.5	**10.8**	30.5
	p-value	0.807	0.446	0.652	0.506	0.229	0.665	0.588	0.937	**0.009**	0.471	0.563	**0.002**	0.862
Insect Powder	Man (%)	**65.4**	70.5	72.4	**60.8**	66.2	29.2	63.2	66.0	**54.9**	41.6	64.4	**56.2**	**39.4**
	Woman (%)	**74.6**	66.5	77.1	**70.3**	71.5	28.6	67.3	66.7	**69.8**	46.3	67.6	**70.5**	**48.6**
	p-value	**0.012**	0.278	0.169	**0.013**	0.153	0.861	0.277	0.866	**≤0.001**	0.229	0.401	**≤0.001**	**0.020**
Lecithin	Man (%)	13.7	35.6	23.5	15.9	22.6	21.9	9.2	**36.8**	39.7	41.0	43.8	**47.9**	33.0
	Woman (%)	18.4	29.4	18.4	13.9	17.7	20.0	6.7	**23.8**	38.4	41.9	48.9	**55.9**	40.0
	p-value	0.104	0.101	0.118	0.482	0.126	0.557	0.239	**≤0.001**	0.744	0.809	0.202	**0.046**	0.069
Malto-dextrins	Man (%)	**33.7**	48.3	47.0	30.9	39.2	3.2	26.3	73.7	6.7	14.6	8.6	15.6	9.2
	Woman (%)	**46.0**	50.3	53.3	33.9	41.5	2.9	28.3	76.5	9.8	18.1	7.3	15.6	7.0
	p-value	**0.002**	0.605	0.111	0.426	0.560	0.817	0.592	0.408	0.148	0.237	0.556	1.000	0.307
Molasses	Man (%)	10.8	27.9	14.3	11.8	11.1	25.7	3.5	35.9	40.6	48.9	**50.8**	**47.0**	44.8
	Woman (%)	15.2	27.2	15.6	16.5	8.5	22.2	2.9	40.0	43.2	53.3	**59.4**	**56.5**	46.0
	p-value	0.098	0.840	0.655	0.093	0.273	0.305	0.650	0.286	0.519	0.265	**0.031**	**0.017**	0.749

Snack food ingredients	Data type	Cluster 1					Cluster 2	Cluster 3	Cluster 4	Cluster 5			Cluster 6	Cluster 7
		**Australia**	**India**	**South Africa**	**UK**[1]	**USA**[1]	**China**	**Japan**	**Russia**	**Brazil**	**Mexico**	**Peru**	**Spain**	**Thailand**
Pea Flour	Man (%)	**4.4**	7.0	7.6	7.6	9.2	3.2	1.9	5.4	2.5	4.4	7.3	11.7	5.7
	Woman (%)	**9.5**	5.1	6.3	4.4	8.9	5.7	1.3	3.2	4.1	4.8	4.8	13.3	3.2
	p-value	**0.012**	0.311	0.532	0.091	0.870	0.122	0.525	0.169	0.268	0.850	0.181	0.548	0.122
Salt	Man (%)	14.0	**11.7**	12.4	12.7	12.7	7.6	2.9	2.9	20.0	12.4	15.2	14.9	9.2
	Woman (%)	14.3	**7.0**	13.3	16.1	11.1	6.0	1.9	4.1	22.5	16.5	15.6	13.0	6.3
	p-value	0.909	**0.039**	0.722	0.225	0.520	0.430	0.434	0.386	0.437	0.141	0.912	0.491	0.181
SAPP	Man (%)	**44.8**	59.0	58.4	38.2	43.0	40.3	29.8	74.0	54.6	60.0	63.5	54.3	61.9
	Woman (%)	**59.4**	56.0	53.7	45.3	47.5	46.7	33.3	75.6	52.1	58.7	66.7	60.0	63.2
	p-value	**≤0.001**	0.441	0.229	0.074	0.260	0.108	0.346	0.647	0.523	0.746	0.404	0.148	0.742
Sodium Bicarbonate	Man (%)	15.6	**40.3**	23.2	11.1	27.1	40.6	19.0	8.6	**35.9**	21.9	**35.6**	27.3	57.5
	Woman (%)	14.0	**31.0**	22.5	11.4	22.5	35.9	18.7	8.3	**25.4**	16.2	**27.0**	23.5	53.0
	p-value	0.575	**0.015**	0.850	0.923	0.181	0.219	0.919	0.886	**0.004**	0.068	**0.020**	0.273	0.262
Sorghum Flour	Man (%)	8.9	14.3	7.0	**15.0**	16.2	4.4	**11.7**	11.7	**12.7**	9.2	12.1	21.3	6.7
	Woman (%)	10.5	9.8	11.4	**8.5**	15.5	6.0	**19.4**	8.6	**19.7**	12.4	13.7	21.0	4.4
	p-value	0.501	0.084	0.054	**0.012**	0.801	0.372	**0.008**	0.188	**0.017**	0.200	0.552	0.923	0.224
Soybeans	Man (%)	8.9	4.4	11.4	10.2	10.8	8.6	1.0	32.7	**4.8**	8.9	**12.7**	11.7	8.3
	Woman (%)	9.2	5.7	15.2	6.0	10.4	8.6	1.0	29.2	**8.9**	7.9	**7.9**	7.6	4.4
	p-value	0.890	0.474	0.160	0.055	0.876	1.000	1.000	0.344	**0.040**	0.667	**0.050**	0.080	0.050
Sugar	Man (%)	19.7	19.0	**17.5**	**15.6**	**20.7**	**15.2**	6.3	**6.7**	15.6	18.4	15.6	23.2	36.8
	Woman (%)	25.1	15.2	**28.6**	**21.8**	**14.6**	**9.8**	7.0	**12.4**	17.1	15.6	14.9	22.9	34.0
	p-value	0.105	0.199	**≤0.001**	**0.045**	**0.043**	**0.041**	0.750	**0.015**	0.591	0.340	0.825	0.925	0.454
Wheat Flour	Man (%)	**3.8**	5.1	**6.0**	5.4	7.3	5.1	2.5	1.6	**7.6**	10.5	5.4	6.7	14.9
	Woman (%)	**9.2**	5.4	**10.5**	5.7	8.5	6.0	2.5	1.6	**16.8**	14.3	8.3	5.1	11.1
	p-value	**0.006**	0.866	**0.043**	0.878	0.572	0.602	1.000	1.000	**≤0.001**	0.147	0.156	0.398	0.156
Xanthan Gum	Man (%)	**28.9**	**55.6**	36.8	30.6	36.6	8.9	18.7	63.5	20.3	15.6	16.5	27.3	26.7
	Woman (%)	**37.1**	**39.9**	40.6	34.5	35.1	6.3	24.4	58.4	25.4	15.6	14.3	32.4	29.5
	p-value	**0.028**	**<0.0001**	0.327	0.294	0.696	0.230	0.082	0.192	0.129	1.000	0.440	0.164	0.426

[1] Country abbreviations, UK=United Kingdom, USA=United States of America

[2] Significant *p*-values (≤ 0.05) are highlighted in bold.

Table S2. Significant differences based on Kruskal-Wallis test in percentage (%) avoidance of ingredients in Snack foods by age for consumers in 13 countries (data shown as 18-34 years (%), 35-54 years (%), 55 years and older (%), and *p*-value).

**Snack food ingredients**	**Data type**	**Cluster 1**					**Cluster 2**	**Cluster 3**	**Cluster 4**	**Cluster 5**			**Cluster 6**	**Cluster 7**
		**Australia**	**India**	**South Africa**	**UK**[1]	**USA**[1]	**China**	**Japan**	**Russia**	**Brazil**	**Mexico**	**Peru**	**Spain**	**Thailand**
Baking Soda	18-34 (%)	**7.1**^{b}[2,3]	34.6	11.4	**9.0**^{a}	9.1	**26.2**^{a}	**6.7**^{b}	**18.1**^{b}	**22.4**^{a}	25.2	37.1	24.3	23.8
	35-54 (%)	**6.2**^{b}	30.0	10.5	**2.9**^{b}	4.3	**10.5**^{b}	**9.5**^{b}	**17.6**^{b}	**13.8**^{b}	18.6	36.2	15.7	26.7
	55+ (%)	**14.3**^{a}	33.8	11.4	**11.0**^{a}	8.6	**14.3**^{b}	**18.6**^{a}	**27.1**^{a}	**13.8**^{b}	16.2	38.1	20.5	29.5
	p-value	**0.007**	0.562	0.938	**0.005**	0.119	**<0.0001**	**≤0.001**	**0.026**	**0.025**	0.056	0.922	0.090	0.417
BHA	18-34 (%)	**41.0**^{b}	50.7	45.7	36.7	**40.7**^{b}	**58.6**^{b}	31.9	**78.6**^{b}	**40.5**^{b}	56.2	57.1	**48.6**^{b}	46.7
	35-54 (%)	**48.1**^{b}	49.0	53.8	39.0	**43.1**^{b}	**71.4**^{a}	32.9	**77.6**^{b}	**52.4**^{a}	59.5	67.1	**62.4**^{a}	48.1
	55+ (%)	**63.3**^{a}	56.2	56.2	47.1	**57.4**^{a}	**50.5**^{b}	39.5	**87.1**^{a}	**53.3**^{a}	61.0	65.2	**67.6**^{a}	54.3
	p-value	**<0.0001**	0.309	0.079	0.073	**≤0.001**	**<0.0001**	0.203	**0.023**	**0.013**	0.596	0.079	**≤0.001**	0.253
Black Beans	18-34 (%)	4.3	8.1	8.1	6.7	8.1	11.4	1.4	6.7	21.9	12.9	27.6	10.0	46.7
	35-54 (%)	4.8	4.8	4.3	5.2	7.7	11.4	1.0	4.8	31.0	12.9	28.6	8.1	47.1
	55+ (%)	7.1	3.8	6.7	7.1	11.0	10.5	0.0	7.1	27.1	11.9	28.1	12.4	51.4
	p-value	0.382	0.136	0.271	0.707	0.431	0.938	0.244	0.564	0.109	0.944	0.977	0.347	0.561
Canola Oil	18-34 (%)	11.4	21.3	**12.4**^{a}	10.5	13.4	7.6	5.2	**25.2**^{c}	11.4	8.1	11.0	**28.1**^{b}	**22.4**^{a}
	35-54 (%)	7.6	15.7	**4.8**^{b}	10.0	9.1	3.3	4.3	**34.8**^{b}	13.3	8.6	10.5	**35.7**^{b}	**11.0**^{b}
	55+ (%)	10.0	14.8	**11.0**^{a}	16.2	7.7	6.2	9.5	**54.8**^{a}	16.7	8.6	14.8	**53.8**^{a}	**17.1**^{ab}
	p-value	0.411	0.157	**0.017**	0.098	0.127	0.157	0.064	**<0.0001**	0.290	0.980	0.336	**<0.0001**	**0.007**
Corn	18-34 (%)	1.9	6.6	4.8	**6.7**^{a}	8.1	**6.7**^{ab}	0.0	1.4	2.4	2.9	3.3	5.7	55.7
	35-54 (%)	2.4	5.7	3.8	**2.4**^{b}	6.2	**2.9**^{b}	1.9	2.9	2.4	1.0	3.3	2.4	61.4
	55+ (%)	3.3	2.4	4.8	**2.9**^{b}	8.1	**8.6**^{a}	1.0	1.9	1.9	1.9	2.4	1.9	60.5
	p-value	0.639	0.105	0.861	**0.048**	0.693	**0.044**	0.133	0.578	0.930	0.361	0.805	0.061	0.443
Corn Syrup	18-34 (%)	**13.3**^{b}	17.1	17.6	12.4	**22.5**^{b}	8.6	5.7	14.3	21.0	**16.7**^{b}	22.4	28.6	**29.5**^{a}
	35-54 (%)	**20.5**^{ab}	13.8	19.5	11.4	**25.8**^{b}	7.1	5.7	11.0	28.1	**22.4**^{ab}	26.2	22.9	**20.5**^{b}
	55+ (%)	**22.9**^{a}	19.5	21.9	19.0	**37.8**^{a}	10.0	6.7	16.2	29.5	**28.1**^{a}	30.0	22.4	**31.0**^{a}
	p-value	**0.035**	0.292	0.543	0.052	**≤0.001**	0.579	0.894	0.290	0.102	**0.019**	0.207	0.262	**0.032**
Gluten	18-34 (%)	11.9	41.2	26.2	**11.9**^{ab}	12.9	2.4	7.6	**48.1**^{b}	**38.1**^{a}	37.6	36.2	13.3	26.7
	35-54 (%)	10.5	34.3	23.8	**6.7**^{b}	16.7	3.8	7.6	**50.0**^{b}	**27.1**^{b}	47.6	36.7	13.3	28.6
	55+ (%)	13.8	45.7	29.5	**14.3**^{a}	21.1	4.3	13.3	**63.3**^{a}	**35.2**^{ab}	47.6	36.2	19.0	35.2
	p-value	0.575	0.056	0.412	**0.038**	0.085	0.543	0.071	**0.003**	**0.048**	0.059	0.993	0.171	0.133
Insect Powder	18-34 (%)	**53.3**^{c}	**65.9**^{b}	**64.8**^{b}	**50.5**^{c}	**52.6**^{b}	**34.8**^{a}	62.9	**61.0**^{b}	**58.1**^{b}	**41.9**^{ab}	60.0	**51.0**^{b}	**37.6**^{b}
	35-54 (%)	**71.0**^{b}	**61.9**^{b}	**70.0**^{b}	**67.1**^{b}	**73.2**^{a}	**30.5**^{a}	64.3	**63.3**^{b}	**56.7**^{b}	**39.0**^{b}	69.0	**65.7**^{a}	**41.4**^{b}
	55+ (%)	**85.7**^{a}	**77.6**^{a}	**89.5**^{a}	**79.0**^{a}	**80.9**^{a}	**21.4**^{b}	68.6	**74.8**^{a}	**72.4**^{a}	**51.0**^{a}	69.0	**73.3**^{a}	**52.9**^{a}
	p-value	**<0.0001**	**0.002**	**<0.0001**	**<0.0001**	**<0.0001**	**0.009**	0.441	**0.006**	**≤0.001**	**0.037**	0.078	**<0.0001**	**0.005**

Snack food ingredients	Data type	Cluster 1					Cluster 2	Cluster 3	Cluster 4	Cluster 5			Cluster 6	Cluster 7
		**Australia**	**India**	**South Africa**	**UK**[1]	**USA**[1]	**China**	**Japan**	**Russia**	**Brazil**	**Mexico**	**Peru**	**Spain**	**Thailand**
Lecithin	18-34 (%)	18.6	**37.4**[a]	24.8	16.2	22.5	**24.3**[a]	9.5	**26.2**[b]	39.0	40.0	41.4	**43.8**[b]	**32.4**[b]
	35-54 (%)	14.8	**24.3**[b]	19.5	12.4	18.7	**24.8**[a]	6.7	**26.7**[b]	37.1	39.0	47.6	**51.0**[b]	**33.3**[b]
	55+ (%)	14.8	**35.7**[a]	18.6	16.2	19.6	**13.8**[b]	7.6	**38.1**[a]	41.0	45.2	50.0	**61.0**[a]	**43.8**[a]
	p-value	0.471	**0.008**	0.245	0.450	0.599	**0.008**	0.545	**0.011**	0.726	0.383	0.192	**0.002**	**0.026**
Maltodextrins	18-34 (%)	**32.4**[b]	**50.2**[ab]	**45.7**[b]	**31.9**[b]	**34.0**[b]	4.8	**22.4**[b]	**73.3**[b]	8.6	17.6	10.5	**14.8**[ab]	**11.9**[a]
	35-54 (%)	**33.8**[b]	**42.4**[b]	**46.7**[b]	**24.3**[b]	**39.2**[ab]	2.4	**26.2**[ab]	**68.6**[b]	7.1	13.3	7.6	**11.4**[b]	**5.2**[b]
	55+ (%)	**53.3**[a]	**55.2**[a]	**58.1**[a]	**41.0**[a]	**48.3**[a]	1.9	**33.3**[a]	**83.3**[a]	9.0	18.1	5.7	**20.5**[a]	**7.1**[ab]
	p-value	**<0.0001**	**0.029**	**0.019**	**≤0.001**	**0.010**	0.186	**0.038**	**0.002**	0.762	0.348	0.192	**0.035**	**0.036**
Molasses	18-34 (%)	15.2	**36.5**[a]	19.5	**18.6**[a]	9.1	**25.2**[ab]	3.3	**31.0**[b]	39.0	46.2	**47.6**[b]	**41.9**[b]	**36.2**[b]
	35-54 (%)	10.0	**20.5**[b]	12.9	**10.0**[b]	8.6	**28.6**[a]	2.9	**33.8**[b]	42.4	50.5	**57.1**[a]	**51.4**[b]	**44.3**[b]
	55+ (%)	13.8	**25.7**[b]	12.4	**13.8**[ab]	12.0	**18.1**[b]	3.3	**49.0**[a]	44.3	56.7	**60.5**[a]	**61.9**[a]	**55.7**[a]
	p-value	0.257	**≤0.001**	0.072	**0.041**	0.464	**0.037**	0.950	**≤0.001**	0.546	0.098	**0.023**	**≤0.001**	**≤0.001**
Pea Flour	18-34 (%)	6.7	**9.0**[a]	7.1	5.7	8.1	6.2	2.4	5.7	4.3	5.7	8.1	10.5	5.2
	35-54 (%)	6.2	**3.3**[b]	5.2	4.3	7.7	3.3	1.0	4.3	1.0	4.3	5.7	11.9	5.2
	55+ (%)	8.1	**5.7**[ab]	8.6	8.1	11.5	3.8	1.4	2.9	4.8	3.8	4.3	15.2	2.9
	p-value	0.728	**0.049**	0.406	0.254	0.333	0.314	0.492	0.352	0.061	0.626	0.254	0.319	0.393
Salt	18-34 (%)	**9.5**[b]	9.0	12.4	10.5	**9.6**[b]	9.0	1.4	**2.4**[b]	**14.3**[b]	12.9	15.2	15.2	9.5
	35-54 (%)	**12.4**[b]	12.4	11.9	14.8	**8.6**[b]	7.1	1.4	**6.2**[a]	**23.3**[a]	12.9	13.3	11.0	8.6
	55+ (%)	**20.5**[a]	6.7	14.3	18.1	**17.7**[a]	4.3	4.3	**1.9**[b]	**26.2**[a]	17.6	17.6	15.7	5.2
	p-value	**0.004**	0.130	0.743	0.084	**0.007**	0.150	0.086	**0.032**	**0.008**	0.277	0.476	0.301	0.228
SAPP	18-34 (%)	**43.8**[b]	58.3	51.9	38.6	**41.1**[b]	**42.9**[b]	**21.9**[c]	**71.0**[b]	48.1	54.8	60.0	**49.5**[b]	**52.9**[b]
	35-54 (%)	**46.7**[b]	54.8	56.2	39.0	**40.7**[b]	**54.8**[a]	**31.0**[b]	**71.9**[b]	56.2	61.4	66.2	**59.0**[a]	**64.8**[a]
	55+ (%)	**65.7**[a]	59.5	60.0	47.6	**54.1**[a]	**32.9**[c]	**41.9**[a]	**81.4**[a]	55.7	61.9	69.0	**62.9**[a]	**70.0**[a]
	p-value	**<0.0001**	0.592	0.248	0.107	**0.008**	**<0.0001**	**<0.0001**	**0.024**	0.176	0.250	0.139	**0.018**	**≤0.001**
Sodium Bicarbonate	18-34 (%)	13.3	39.8	25.7	**16.2**[a]	25.4	38.1	**13.3**[b]	8.1	28.6	21.0	34.3	**31.4**[a]	**48.6**[b]
	35-54 (%)	12.4	30.5	17.1	**7.6**[b]	24.9	41.0	**16.7**[b]	7.6	32.4	20.0	29.0	**20.5**[b]	**52.9**[b]
	55+ (%)	18.6	36.7	25.7	**10.0**[b]	24.4	35.7	**26.7**[a]	9.5	31.0	16.2	30.5	**24.3**[ab]	**64.3**[a]
	p-value	0.157	0.127	0.054	**0.016**	0.975	0.543	**≤0.001**	0.765	0.694	0.422	0.489	**0.033**	**0.004**
Sorghum Flour	18-34 (%)	10.5	13.7	10.5	**14.3**[a]	16.3	5.2	**13.8**[b]	10.0	12.9	11.4	11.9	**14.3**[b]	**9.0**[a]
	35-54 (%)	7.6	8.6	6.2	**7.1**[b]	19.1	4.3	**11.4**[b]	7.6	14.8	8.1	9.5	**22.4**[a]	**3.8**[b]
	55+ (%)	11.0	13.8	11.0	**13.8**[a]	12.4	6.2	**21.4**[a]	12.9	21.0	12.9	17.1	**26.7**[a]	**3.8**[b]
	p-value	0.459	0.167	0.178	**0.040**	0.172	0.682	**0.013**	0.206	0.063	0.272	0.058	**0.007**	**0.026**
Soybeans	18-34 (%)	**4.8**[b]	7.6	**9.0**[b]	7.1	12.0	**7.1**[b]	1.4	30.0	6.7	10.5	10.5	8.1	**7.6**[a]
	35-54 (%)	**10.5**[a]	4.8	**11.4**[b]	7.1	10.0	**5.2**[b]	1.4	29.5	4.8	4.8	10.5	8.6	**8.6**[a]
	55+ (%)	**11.9**[a]	2.9	**19.5**[a]	10.0	9.6	**13.3**[a]	0.0	33.3	9.0	10.0	10.0	12.4	**2.9**[b]
	p-value	**0.026**	0.085	**0.004**	0.464	0.701	**0.008**	0.220	0.655	0.219	0.065	0.983	0.266	**0.037**

Snack food ingredients	Data type	Cluster 1					Cluster 2	Cluster 3	Cluster 4	Cluster 5			Cluster 6	Cluster 7
		**Australia**	**India**	**South Africa**	**UK**[1]	**USA**[1]	**China**	**Japan**	**Russia**	**Brazil**	**Mexico**	**Peru**	**Spain**	**Thailand**
Sugar	18-34 (%)	20.0	15.6	22.9	18.1	**12.4**[b]	10.5	**2.4**[b]	12.4	20.0	17.6	14.8	23.8	**29.5**[b]
	35-54 (%)	20.5	16.7	18.6	14.8	**15.8**[b]	12.4	**6.7**[ab]	7.1	15.7	16.2	17.1	19.0	**31.0**[b]
	55+ (%)	26.7	19.0	27.6	23.3	**24.4**[a]	14.8	**11.0**[a]	9.0	13.3	17.1	13.8	26.2	**45.7**[a]
	p-value	0.188	0.636	0.089	0.076	**0.004**	0.414	**0.002**	0.181	0.174	0.924	0.620	0.209	**≤0.001**
Wheat Flour	18-34 (%)	8.1	6.6	6.7	7.6	9.1	6.2	3.3	1.9	11.0	11.4	5.7	7.6	**17.6**[a]
	35-54 (%)	5.7	5.7	7.6	4.8	7.2	5.2	2.9	1.9	12.4	12.4	6.7	6.2	**11.4**[ab]
	55+ (%)	5.7	3.3	10.5	4.3	7.2	5.2	1.4	1.0	13.3	13.3	8.1	3.8	**10.0**[b]
	p-value	0.521	0.292	0.337	0.273	0.702	0.886	0.435	0.666	0.755	0.839	0.623	0.245	**0.048**
Xanthan Gum	18-34 (%)	**24.8**[b]	47.4	**33.8**[b]	**26.2**[b]	32.1	9.5	**17.1**[b]	**58.1**[b]	27.1	14.8	15.7	25.7	**20.0**[b]
	35-54 (%)	**30.5**[b]	42.9	**37.1**[ab]	**31.4**[ab]	34.4	8.1	**20.5**[ab]	**54.3**[b]	21.9	13.3	14.3	29.0	**18.1**[b]
	55+ (%)	**43.8**[a]	52.9	**45.2**[a]	**40.0**[a]	41.6	5.2	**27.1**[a]	**70.5**[a]	19.5	18.6	16.2	34.8	**46.2**[a]
	p-value	**≤0.001**	0.122	**0.047**	**0.010**	0.106	0.242	**0.040**	**0.002**	0.164	0.310	0.854	0.123	**<0.0001**

[1] Country abbreviations, UK=United Kingdom, USA=United States of America

[2] Significant *p*-values (≤ 0.05) are highlighted in bold.

[3] [a,b,c] letters indicate significant differences between ages of each snack food ingredient. Post hoc pairwise comparisons analysis was conducted using the critical difference (Dunn) procedure.

Table S3. Significant differences based on Mann-Whitney U test in percentage (%) avoidance of ingredients in Snack foods by education level for consumers in 13 countries (data shown as High school or less (%), College or university graduates (%), and *p*-value).

Snack food ingredients	Data type	Cluster 1					Cluster 2	Cluster 3	Cluster 4	Cluster 5			Cluster 6	Cluster 7
		**Australia**	**India**	**South Africa**	**UK[1]**	**USA[1]**	**China**	**Japan**	**Russia**	**Brazil**	**Mexico**	**Peru**	**Spain**	**Thailand**
Baking Soda	HS or less (%)	9.6[2]	33.3	9.5	7.3	6.8	20.1	14.0	26.8	16.6	**35.7**	45.9	18.6	24.4
	College+ (%)	9.0	32.8	12.0	7.8	7.7	16.1	10.3	20.4	16.7	**19.3**	36.2	21.6	27.0
	p-value	0.802	0.950	0.335	0.838	0.653	0.272	0.170	0.262	0.971	**0.034**	0.137	0.353	0.607
BHA	HS or less (%)	53.4	53.3	51.9	41.6	50.3	**50.7**	36.5	73.2	50.2	**78.6**	57.4	60.1	44.4
	College+ (%)	49.1	51.9	51.9	40.5	44.0	**62.7**	33.8	81.9	47.7	**58.0**	63.8	59.0	50.6
	p-value	0.290	0.880	0.987	0.782	0.115	**0.012**	0.503	0.114	0.540	**0.031**	0.324	0.769	0.284
Black Beans	HS or less (%)	**8.0**	13.3	7.4	8.2	10.9	12.7	0.9	8.9	27.4	21.4	34.4	10.5	46.7
	College+ (%)	**3.7**	5.2	5.8	5.2	7.1	10.7	0.7	5.9	26.1	12.1	27.4	9.9	48.7
	p-value	**0.020**	0.057	0.430	0.137	0.100	0.514	0.824	0.374	0.724	0.147	0.248	0.806	0.721
Canola Oil	HS or less (%)	8.8	20.0	9.5	13.1	10.9	8.2	7.2	41.1	11.2	3.6	19.7	**43.9**	21.1
	College+ (%)	10.3	17.1	9.3	11.7	9.5	5.0	5.9	38.0	15.6	8.6	11.2	**35.0**	16.1
	p-value	0.527	0.687	0.918	0.609	0.573	0.161	0.515	0.650	0.113	0.346	0.055	**0.023**	0.241
Corn	HS or less (%)	2.0	0.0	3.0	3.3	7.1	7.5	0.9	5.4	2.7	0.0	4.9	3.4	54.4
	College+ (%)	2.9	5.2	5.3	4.4	8.0	5.6	1.0	1.7	1.9	2.0	2.8	3.3	60.0
	p-value	0.478	0.203	0.191	0.472	0.674	0.434	0.923	0.070	0.495	0.452	0.362	0.953	0.321
Corn Syrup	HS or less (%)	17.1	20.0	**14.7**	17.6	29.6	9.7	5.0	21.4	**21.2**	14.3	31.1	23.0	26.7
	College+ (%)	20.1	16.6	**22.6**	12.2	28.3	8.3	6.6	13.1	**29.6**	22.8	25.7	26.0	27.0
	p-value	0.360	0.632	**0.017**	0.062	0.716	0.599	0.403	0.084	**0.018**	0.294	0.355	0.372	0.942
Gluten	HS or less (%)	**15.5**	40.0	22.5	11.0	18.0	2.2	9.5	55.4	36.3	46.4	39.3	14.9	31.1
	College+ (%)	**9.8**	40.4	28.8	10.9	16.1	3.8	9.6	53.7	31.5	44.2	36.0	15.6	30.0
	p-value	**0.029**	0.963	0.084	0.966	0.515	0.374	0.968	0.808	0.214	0.816	0.609	0.807	0.832
Insect Powder	HS or less (%)	**74.9**	53.3	74.9	**71.0**	72.1	22.4	63.1	69.6	62.5	57.1	72.1	65.9	47.8
	College+ (%)	**66.8**	69.2	74.7	**62.1**	66.1	30.6	66.4	66.0	62.3	43.4	65.4	61.1	43.3
	p-value	**0.029**	0.068	0.955	**0.021**	0.103	0.062	0.398	0.585	0.943	0.151	0.290	0.213	0.432
Lecithin	HS or less (%)	19.1	40.0	20.8	15.1	23.5	**10.4**	8.1	41.1	40.9	**60.7**	47.5	52.0	32.2
	College+ (%)	14.0	32.1	21.1	14.8	17.3	**23.8**	7.8	29.3	37.7	**40.5**	46.2	51.8	37.2
	p-value	0.086	0.369	0.936	0.919	0.053	**≤0.001**	0.907	0.067	0.420	**0.034**	0.845	0.954	0.362
Maltodextrins	HS or less (%)	44.2	56.7	48.5	33.5	41.8	0.7	27.5	67.9	7.7	21.4	13.1	17.6	7.8
	College+ (%)	36.9	48.9	51.1	31.7	39.0	3.6	27.2	75.8	8.6	16.1	7.4	13.8	8.1
	p-value	0.068	0.408	0.523	0.642	0.468	0.084	0.942	0.191	0.686	0.458	0.116	0.190	0.906
Molasses	HS or less (%)	12.7	36.7	14.3	15.9	7.8	**14.2**	3.2	48.2	42.1	**71.4**	57.4	55.4	45.6
	College+ (%)	13.2	27.1	15.3	13.0	11.6	**26.6**	3.2	36.9	41.8	**50.2**	54.8	48.5	45.4
	p-value	0.872	0.254	0.734	0.304	0.112	**0.003**	0.983	0.097	0.939	**0.028**	0.705	0.084	0.974

Snack food ingredients	Data type	Cluster 1					Cluster 2	Cluster 3	Cluster 4	Cluster 5			Cluster 6	Cluster 7
		**Australia**	**India**	**South Africa**	**UK[1]**	**USA[1]**	**China**	**Japan**	**Russia**	**Brazil**	**Mexico**	**Peru**	**Spain**	**Thailand**
Pea Flour	HS or less (%)	8.4	**20.0**	8.2	8.2	**11.6**	5.2	2.3	**16.1**	2.7	3.6	9.8	**16.6**	2.2
	College+ (%)	6.1	**5.3**	6.3	4.7	**6.8**	4.2	1.2	**3.1**	3.8	4.7	5.6	**9.0**	4.8
	p-value	0.269	**≤0.001**	0.353	0.073	**0.040**	0.623	0.326	**<0.0001**	0.462	0.791	0.190	**0.004**	0.270
Salt	HS or less (%)	14.3	6.7	11.3	16.3	12.6	6.0	3.2	3.6	**17.4**	14.3	18.0	12.5	7.8
	College+ (%)	14.0	9.5	13.8	13.2	11.3	7.1	2.0	3.5	**24.0**	14.5	15.1	15.3	7.8
	p-value	0.900	0.606	0.361	0.284	0.622	0.659	0.349	0.974	**0.046**	0.981	0.549	0.317	1.000
SAPP	HS or less (%)	56.2	50.0	54.5	40.0	48.3	36.6	33.3	76.8	52.5	75.0	65.6	59.5	**52.2**
	College+ (%)	49.3	57.9	56.9	42.9	42.6	45.4	30.6	74.6	53.9	58.6	65.0	55.1	**64.3**
	p-value	0.093	0.393	0.568	0.479	0.149	0.069	0.487	0.716	0.730	0.085	0.932	0.269	**0.029**
Sodium Bicarbonate	HS or less (%)	17.9	30.0	22.5	9.4	**30.3**	**47.8**	**24.3**	12.5	**35.1**	25.0	39.3	26.0	48.9
	College+ (%)	12.7	35.9	23.1	12.5	**19.9**	**35.7**	**15.9**	8.0	**27.5**	18.8	30.4	24.9	56.3
	p-value	0.069	0.508	0.875	0.234	**0.003**	**0.011**	**0.010**	0.249	**0.041**	0.413	0.153	0.738	0.191
Sorghum Flour	HS or less (%)	**13.1**	**26.7**	10.8	13.5	18.7	**9.7**	16.2	14.3	17.8	**28.6**	**21.3**	**25.3**	7.8
	College+ (%)	**7.4**	**11.3**	8.3	10.6	13.4	**4.0**	15.2	9.8	15.1	**10.0**	**12.0**	**17.4**	5.2
	p-value	**0.017**	**0.012**	0.286	0.284	0.069	**0.009**	0.736	0.285	0.372	**0.002**	**0.038**	**0.014**	0.321
Soybeans	HS or less (%)	8.0	3.3	15.2	7.8	**14.3**	**15.7**	0.5	39.3	6.9	**21.4**	14.8	10.1	5.6
	College+ (%)	9.8	5.2	12.3	8.3	**7.4**	**6.7**	1.2	30.1	6.7	**7.8**	9.8	9.3	6.5
	p-value	0.443	0.658	0.308	0.803	**0.005**	**≤0.001**	0.340	0.158	0.918	**0.011**	0.231	0.718	0.740
Sugar	HS or less (%)	21.9	16.7	19.0	20.0	**13.9**	15.7	7.2	12.5	18.5	21.4	18.0	25.0	28.9
	College+ (%)	22.7	17.1	25.3	17.9	**20.8**	11.7	6.4	9.2	14.8	16.8	14.9	21.3	36.5
	p-value	0.819	0.947	0.072	0.515	**0.024**	0.218	0.689	0.427	0.216	0.523	0.523	0.266	0.164
Wheat Flour	HS or less (%)	5.6	0.0	**5.2**	5.7	7.8	7.5	1.8	3.6	11.2	7.1	**14.8**	6.1	12.2
	College+ (%)	7.1	5.5	**10.0**	5.5	8.0	5.0	2.9	1.4	12.9	12.6	**6.0**	5.7	13.1
	p-value	0.442	0.188	**0.034**	0.890	0.922	0.278	0.386	0.214	0.512	0.390	**0.010**	0.835	0.810
Xanthan Gum	HS or less (%)	36.7	**73.3**	40.7	35.9	38.1	5.2	23.0	64.3	23.6	14.3	21.3	32.1	23.3
	College+ (%)	30.6	**46.4**	37.6	30.4	33.9	8.3	20.8	60.6	22.4	15.6	14.8	27.8	28.9
	p-value	0.114	**0.004**	0.442	0.149	0.277	0.239	0.533	0.593	0.729	0.850	0.179	0.245	0.278

[1] Country abbreviations, UK=United Kingdom, USA=United States of America

[2] Significant *p*-values (≤ 0.05) are highlighted in bold.

Table S4. Significant differences based on Mann-Whitney U test in percentage (%) avoidance of ingredients in Snack foods by number of adults in household for consumers in 13 countries (data shown as households with 1-2 adults (%), households with 3 or more adults (%), and *p*-value).

Snack food ingredients	Data type	Cluster 1					Cluster 2	Cluster 3	Cluster 4	Cluster 5			Cluster 6	Cluster 7
		Australia	India	South Africa	UK[1]	USA[1]	China	Japan	Russia	Brazil	Mexico	Peru	Spain	Thailand
Baking Soda	1-2 (%)	9.8[2]	33.5	10.7	7.6	7.5	16.8	12.0	20.2	17.7	10.3	30.8	27.0	26.2
	3+ (%)	7.2	32.5	11.7	7.7	6.8	17.0	11.1	22.3	15.6	20.6	37.4	19.4	26.9
	p-value	0.336	0.813	0.690	0.948	0.791	0.942	0.747	0.539	0.476	0.117	0.493	0.155	0.855
BHA	1-2 (%)	**53.1**	51.4	53.2	42.5	47.8	60.8	35.3	81.8	51.5	**43.6**	**30.8**	63.5	44.5
	3+ (%)	**43.4**	52.2	50.2	36.1	44.7	60.0	34.1	79.9	45.7	**59.9**	**64.6**	59.1	51.9
	p-value	**0.037**	0.869	0.463	0.160	0.505	0.850	0.751	0.562	0.144	**0.045**	**≤0.001**	0.499	0.087
Black Beans	1-2 (%)	5.2	3.5	5.8	6.5	8.7	8.4	0.9	6.5	28.7	5.1	23.1	9.5	**41.9**
	3+ (%)	5.9	6.3	7.2	5.8	9.3	11.9	0.7	5.7	24.5	13.0	28.3	10.2	**51.3**
	p-value	0.743	0.161	0.472	0.750	0.826	0.240	0.755	0.687	0.239	0.150	0.562	0.861	**0.031**
Canola Oil	1-2 (%)	9.4	16.8	10.4	12.8	11.5	2.8	6.3	40.4	15.2	12.8	11.5	47.6	18.3
	3+ (%)	10.5	17.5	7.9	10.3	6.2	6.6	6.4	34.5	12.3	8.1	12.1	38.3	16.2
	p-value	0.687	0.835	0.291	0.406	0.055	0.088	0.947	0.143	0.277	0.307	0.934	0.150	0.508
Corn	1-2 (%)	3.1	5.2	4.7	4.4	7.0	3.5	0.6	1.7	1.8	0.0	**11.5**	**7.9**	**49.2**
	3+ (%)	0.7	4.8	4.2	2.6	9.3	6.8	1.4	2.6	2.6	2.0	**2.6**	**2.8**	**63.6**
	p-value	0.091	0.837	0.761	0.309	0.347	0.148	0.333	0.459	0.486	0.370	**0.010**	**0.032**	**≤0.001**
Corn Syrup	1-2 (%)	20.5	20.8	21.1	14.9	29.9	5.6	5.7	15.7	**29.6**	25.6	**53.8**	20.6	**36.1**
	3+ (%)	13.8	15.3	17.7	12.3	26.1	9.4	6.4	10.5	**22.5**	22.2	**25.0**	25.0	**23.0**
	p-value	0.067	0.098	0.296	0.407	0.364	0.149	0.701	0.067	**0.044**	0.615	**≤0.001**	0.441	**≤0.001**
Gluten	1-2 (%)	**13.6**	**48.0**	27.1	11.6	17.9	2.1	9.0	54.4	32.3	43.6	26.9	19.0	25.7
	3+ (%)	**7.2**	**37.6**	25.7	9.0	14.3	3.9	10.1	52.8	34.8	44.3	36.8	14.8	32.1
	p-value	**0.036**	**0.017**	0.682	0.379	0.291	0.302	0.623	0.712	0.515	0.928	0.308	0.376	0.105
Insect Powder	1-2 (%)	**72.4**	**78.6**	76.7	66.3	70.4	23.8	**68.9**	65.3	61.9	48.7	53.8	66.7	**36.1**
	3+ (%)	**62.5**	**64.6**	72.1	63.2	64.6	30.4	**61.1**	68.1	62.9	43.7	66.6	63.0	**47.4**
	p-value	**0.021**	**≤0.001**	0.186	0.483	0.173	0.125	**0.043**	0.477	0.791	0.538	0.181	0.563	**0.009**
Lecithin	1-2 (%)	16.5	32.9	18.4	15.4	20.0	19.6	8.1	27.9	**43.9**	35.9	30.8	58.7	**28.8**
	3+ (%)	14.5	32.3	24.5	13.5	20.5	21.4	7.8	34.5	**33.8**	41.8	47.0	51.1	**39.9**
	p-value	0.548	0.880	0.060	0.581	0.902	0.647	0.885	0.085	**0.009**	0.470	0.104	0.254	**0.008**
Maltodextrins	1-2 (%)	41.4	51.4	51.2	32.2	41.2	2.8	25.4	74.3	8.8	15.4	15.4	20.6	**14.7**
	3+ (%)	34.9	48.5	48.7	32.9	37.9	3.1	29.4	76.4	7.6	16.4	7.6	15.0	**5.2**
	p-value	0.151	0.506	0.527	0.873	0.467	0.863	0.268	0.557	0.577	0.867	0.152	0.242	**<0.0001**
Molasses	1-2 (%)	13.0	25.4	**12.1**	14.7	10.2	21.7	2.7	37.9	**45.7**	**35.9**	**34.6**	57.1	**34.0**
	3+ (%)	13.2	28.4	**18.9**	12.3	8.7	24.6	3.7	38.0	**37.7**	**52.1**	**56.0**	51.1	**50.3**
	p-value	0.953	0.460	**0.018**	0.442	0.572	0.466	0.466	0.983	**0.043**	**0.050**	**0.032**	0.367	**≤0.001**

Snack food ingredients	Data type	Cluster 1					Cluster 2	Cluster 3	Cluster 4	Cluster 5			Cluster 6	Cluster 7
		**Australia**	**India**	**South Africa**	**UK[1]**	**USA[1]**	**China**	**Japan**	**Russia**	**Brazil**	**Mexico**	**Peru**	**Spain**	**Thailand**
Pea Flour	1-2 (%)	7.5	4.6	7.4	6.5	9.0	2.8	**0.6**	3.7	2.7	5.1	11.5	15.9	**8.9**
	3+ (%)	5.3	6.6	6.4	4.5	9.3	4.9	**2.7**	5.2	4.0	4.6	5.8	12.2	**2.5**
	p-value	0.340	0.365	0.634	0.362	0.891	0.278	**0.035**	0.372	0.391	0.873	0.229	0.400	**≤0.001**
Salt	1-2 (%)	14.2	8.1	13.2	14.7	12.2	**2.8**	2.4	3.0	22.3	23.1	**34.6**	17.5	**16.8**
	3+ (%)	13.8	9.8	12.5	13.5	11.2	**8.0**	2.4	4.4	20.2	13.9	**14.6**	13.6	**3.9**
	p-value	0.900	0.505	0.797	0.715	0.743	**0.030**	0.981	0.367	0.529	0.114	**0.006**	0.400	**<0.0001**
SAPP	1-2 (%)	**54.4**	63.0	57.0	42.9	46.1	**35.0**	29.0	73.6	55.8	56.4	**34.6**	61.9	**55.0**
	3+ (%)	**44.7**	55.5	54.7	38.1	42.9	**46.0**	34.5	76.9	50.7	59.6	**66.4**	56.6	**65.8**
	p-value	**0.038**	0.087	0.572	0.285	0.482	**0.019**	0.145	0.361	0.198	0.699	**≤0.001**	0.421	**0.010**
Sodium Bicarbonate	1-2 (%)	13.8	33.5	23.3	10.3	25.2	37.1	18.0	9.0	32.3	15.4	19.2	22.2	**46.6**
	3+ (%)	17.8	36.5	22.3	14.2	23.6	38.6	19.9	7.4	28.8	19.3	31.8	25.7	**59.0**
	p-value	0.232	0.493	0.763	0.185	0.693	0.739	0.529	0.500	0.340	0.548	0.177	0.542	**0.004**
Sorghum Flour	1-2 (%)	9.2	8.7	10.1	**13.3**	14.3	3.5	14.7	10.0	18.3	12.8	15.4	25.4	**8.9**
	3+ (%)	11.2	13.3	7.9	**7.1**	20.5	5.7	16.6	10.5	13.9	10.7	12.7	20.6	**4.1**
	p-value	0.473	0.110	0.344	**0.039**	0.063	0.288	0.516	0.840	0.136	0.674	0.695	0.380	**0.016**
Soybeans	1-2 (%)	9.0	3.5	15.1	9.3	9.8	4.9	0.9	29.2	5.2	12.8	15.4	**17.5**	**11.5**
	3+ (%)	9.2	5.7	10.9	4.5	13.0	9.7	1.0	34.1	8.6	8.1	10.1	**8.8**	**4.1**
	p-value	0.936	0.260	0.133	0.060	0.251	0.074	0.883	0.203	0.089	0.307	0.387	**0.028**	**≤0.001**
Sugar	1-2 (%)	23.0	21.4	**26.6**	19.8	18.3	13.3	7.2	11.0	17.7	20.5	7.7	25.4	34.6
	3+ (%)	20.4	15.5	**18.1**	15.5	15.5	12.3	6.1	7.0	14.9	16.8	15.6	22.8	35.8
	p-value	0.501	0.080	**0.013**	0.233	0.420	0.760	0.580	0.102	0.346	0.545	0.275	0.637	0.771
Wheat Flour	1-2 (%)	7.1	4.6	**10.4**	5.7	7.9	2.8	2.1	1.5	11.9	15.4	**19.2**	4.8	**25.1**
	3+ (%)	4.6	5.5	**5.3**	5.2	8.1	6.4	3.0	1.7	12.6	12.2	**6.3**	6.0	**7.7**
	p-value	0.276	0.675	**0.021**	0.806	0.941	0.102	0.453	0.810	0.791	0.557	**0.011**	0.693	**<0.0001**
Xanthan Gum	1-2 (%)	**35.6**	48.6	38.9	33.7	36.5	4.9	22.5	61.1	**26.2**	10.3	7.7	30.2	29.3
	3+ (%)	**25.0**	47.4	38.5	29.0	34.2	8.4	20.6	60.7	**19.2**	15.9	15.7	29.8	27.6
	p-value	**0.016**	0.792	0.917	0.284	0.600	0.163	0.574	0.922	**0.036**	0.347	0.267	0.954	0.653

[1] Country abbreviations, UK=United Kingdom, USA=United States of America

[2] Significant *p*-values (≤ 0.05) are highlighted in bold.

Table S5. Significant differences based on Mann-Whitney U test in percentage (%) avoidance of ingredients in Snack foods by presence of children in household for consumers in 13 countries (data shown as households without children (%), households with children (%), and *p*-value).

Snack food ingredients	Data type	Cluster 1					Cluster 2	Cluster 3	Cluster 4	Cluster 5			Cluster 6	Cluster 7
		**Australia**	**India**	**South Africa**	**UK**[1]	**USA**[1]	**China**	**Japan**	**Russia**	**Brazil**	**Mexico**	**Peru**	**Spain**	**Thailand**
Baking Soda	none (%)	10.0[2]	31.8	11.5	7.7	7.5	13.8	12.1	23.3	14.6	**15.1**	40.7	19.8	**22.9**
	any (%)	7.6	33.5	10.7	7.4	6.8	19.3	10.2	18.4	19.2	**23.0**	34.8	20.6	**30.1**
	p-value	0.318	0.647	0.735	0.889	0.735	0.068	0.529	0.132	0.124	**0.017**	0.134	0.812	**0.042**
BHA	none (%)	**55.6**	**58.4**	52.2	**43.9**	**52.6**	64.6	35.3	**84.7**	49.0	57.6	62.9	61.1	52.8
	any (%)	**41.2**	**47.6**	51.5	**35.8**	**35.4**	56.9	33.1	**77.3**	48.4	59.7	63.4	57.3	46.8
	p-value	**≤0.001**	**0.008**	0.868	**0.047**	**<0.0001**	0.053	0.619	**0.018**	0.882	0.599	0.910	0.327	0.132
Black Beans	none (%)	5.7	4.7	6.8	6.2	9.2	**7.5**	0.6	7.7	26.9	10.1	27.4	9.8	45.8
	any (%)	4.7	6.1	5.8	6.6	8.3	**13.8**	1.3	4.6	26.3	14.0	28.5	10.7	50.8
	p-value	0.605	0.448	0.629	0.876	0.696	**0.012**	0.435	0.111	0.866	0.147	0.761	0.712	0.218
Canola Oil	none (%)	10.3	18.8	10.3	12.7	9.0	**3.0**	6.6	**43.6**	14.9	10.5	12.5	**43.2**	17.3
	any (%)	8.5	16.2	8.2	11.4	12.6	**7.7**	5.7	**32.6**	12.5	7.1	11.8	**33.6**	16.4
	p-value	0.488	0.397	0.373	0.616	0.154	**0.011**	0.715	**0.005**	0.377	0.141	0.787	**0.015**	0.773
Corn	none (%)	2.9	3.9	4.1	3.5	7.3	5.2	0.8	1.2	2.6	1.3	1.6	**1.9**	58.8
	any (%)	1.9	5.6	4.8	4.8	8.3	6.6	1.3	3.0	1.8	2.3	3.9	**5.3**	59.6
	p-value	0.467	0.343	0.680	0.418	0.677	0.464	0.634	0.127	0.500	0.358	0.098	**0.018**	0.844
Corn Syrup	none (%)	21.0	19.6	20.6	**16.5**	**32.3**	7.8	6.1	16.3	25.5	25.6	**31.9**	24.5	26.9
	any (%)	14.7	14.9	18.6	**10.5**	**21.8**	9.1	5.7	11.2	27.0	20.4	**22.5**	24.8	27.1
	p-value	0.056	0.120	0.511	**0.039**	**0.007**	0.571	0.856	0.065	0.662	0.128	**0.009**	0.920	0.968
Gluten	none (%)	12.2	**47.1**	27.7	10.5	17.9	2.6	9.7	**58.3**	32.4	41.2	39.1	14.7	30.6
	any (%)	11.8	**35.9**	25.1	11.8	15.0	4.1	8.9	**49.0**	34.9	46.2	34.6	16.0	29.8
	p-value	0.907	**0.005**	0.454	0.611	0.368	0.301	0.766	**0.020**	0.510	0.221	0.246	0.641	0.832
Insect Powder	none (%)	**76.1**	**75.3**	**82.0**	**73.1**	**71.5**	29.9	**67.7**	**70.9**	65.3	44.5	66.5	65.5	**51.2**
	any (%)	**57.8**	**63.8**	**66.3**	**52.4**	**63.6**	28.2	**58.0**	**61.5**	58.7	43.6	65.7	60.3	**37.4**
	p-value	**<0.0001**	**0.002**	**<0.0001**	**<0.0001**	**0.046**	0.647	**0.027**	**0.013**	0.089	0.823	0.831	0.184	**≤0.001**
Lecithin	none (%)	14.1	**37.6**	**18.0**	15.5	20.3	19.0	6.8	31.0	39.5	41.2	50.8	53.0	38.5
	any (%)	19.9	**29.0**	**24.4**	14.0	19.9	22.4	11.5	29.6	38.4	41.6	43.5	50.4	34.7
	p-value	0.060	**0.023**	**0.049**	0.615	0.912	0.308	0.059	0.708	0.777	0.921	0.071	0.519	0.312
Maltodextrins	none (%)	**44.2**	49.8	52.5	34.4	42.5	2.6	26.0	**78.5**	9.5	15.1	6.5	16.6	6.6
	any (%)	**31.3**	48.9	47.4	28.8	35.9	3.3	31.2	**71.4**	6.8	17.1	8.9	14.1	9.4

Snack food ingredients	Data type	Cluster 1					Cluster 2	Cluster 3	Cluster 4	Cluster 5			Cluster 6	Cluster 7
		**Australia**	**India**	**South Africa**	**UK[1]**	**USA[1]**	**China**	**Japan**	**Russia**	**Brazil**	**Mexico**	**Peru**	**Spain**	**Thailand**
	p-value	**0.002**	0.831	0.204	0.149	0.117	0.611	0.205	**0.038**	0.222	0.518	0.267	0.403	0.202
Molasses	none (%)	13.6	29.4	13.3	13.5	10.8	22.0	3.0	**42.0**	42.4	52.1	59.7	53.3	47.5
	any (%)	11.8	26.3	16.8	15.3	7.8	25.4	3.8	**33.6**	41.3	50.5	52.1	49.6	43.5
	p-value	0.537	0.396	0.211	0.529	0.224	0.324	0.594	**0.029**	0.776	0.699	0.062	0.368	0.309
Pea Flour	none (%)	7.2	5.9	8.0	5.2	9.0	**2.2**	1.5	4.3	2.9	2.9	4.0	13.0	3.7
	any (%)	6.6	6.1	5.8	7.4	9.2	**6.1**	1.9	4.3	3.9	5.6	7.3	11.8	5.2
	p-value	0.808	0.904	0.298	0.268	0.915	**0.021**	0.709	0.992	0.467	0.121	0.090	0.651	0.358
Salt	none (%)	**16.9**	9.0	11.8	14.2	12.3	**3.4**	3.0	3.7	22.6	14.3	**19.8**	14.4	5.6
	any (%)	**8.5**	9.6	14.1	14.8	11.2	**9.4**	0.6	3.3	19.6	14.5	**12.6**	13.4	9.7
	p-value	**0.004**	0.815	0.393	0.828	0.690	**0.003**	0.099	0.790	0.351	0.930	**0.015**	0.710	0.056
SAPP	none (%)	**57.3**	60.4	58.1	44.4	**48.3**	40.3	31.9	76.7	53.0	58.0	67.7	57.9	63.8
	any (%)	**41.7**	55.6	53.6	37.1	**38.8**	45.9	30.6	72.7	53.7	60.2	63.4	56.1	61.4
	p-value	**≤0.001**	0.231	0.257	0.075	**0.025**	0.165	0.753	0.250	0.856	0.583	0.259	0.658	0.536
Sodium	none (%)	16.7	34.9	23.3	10.2	23.1	36.9	19.0	9.5	30.1	15.1	31.9	28.3	57.5
Bicarbonate	any (%)	10.9	36.2	22.3	13.1	28.2	39.2	18.5	7.2	31.3	21.4	30.9	21.4	53.2
	p-value	0.053	0.745	0.774	0.273	0.169	0.560	0.878	0.305	0.739	0.051	0.799	0.051	0.281
Sorghum Flour	none (%)	9.3	14.9	10.0	12.2	15.3	**1.1**	16.5	11.7	**20.1**	12.6	**16.1**	20.4	**3.3**
	any (%)	10.4	10.1	8.2	10.9	17.0	**8.3**	12.7	8.6	**11.4**	9.7	**10.7**	22.1	**7.6**
	p-value	0.655	0.070	0.441	0.626	0.593	**<0.0001**	0.262	0.198	**0.003**	0.254	**0.048**	0.595	**0.019**
Soybeans	none (%)	9.3	4.7	**16.2**	8.7	10.6	**5.6**	0.6	30.7	**9.7**	9.7	12.1	9.5	**3.7**
	any (%)	8.5	5.3	**10.0**	7.0	10.7	**10.8**	1.9	31.3	**3.2**	7.7	9.2	9.9	**8.8**
	p-value	0.749	0.731	**0.021**	0.442	0.980	**0.022**	0.154	0.876	**≤0.001**	0.379	0.237	0.863	**0.008**
Sugar	none (%)	**24.8**	20.4	23.6	19.2	18.9	**15.7**	7.4	11.0	14.0	19.3	15.3	23.1	37.5
	any (%)	**17.5**	14.9	22.3	17.9	15.0	**10.2**	4.5	7.9	19.2	15.6	15.2	22.9	33.4
	p-value	**0.039**	0.072	0.708	0.688	0.238	**0.041**	0.201	0.179	0.081	0.223	0.962	0.954	0.282
Wheat Flour	none (%)	6.0	3.9	10.0	5.0	7.1	**3.4**	2.5	1.8	12.0	14.3	7.7	5.2	12.3
	any (%)	7.6	6.1	6.2	6.6	9.7	**7.2**	2.5	1.3	12.5	11.2	6.3	6.9	13.7
	p-value	0.438	0.225	0.081	0.411	0.252	**0.038**	0.995	0.600	0.873	0.259	0.503	0.370	0.606
Xanthan Gum	none (%)	**35.8**	51.8	39.5	34.7	38.2	6.7	20.9	62.6	22.1	15.5	17.7	31.0	29.2
	any (%)	**27.5**	44.9	37.8	28.8	31.1	8.3	23.6	59.2	23.8	15.6	13.9	28.2	27.1
	p-value	**0.036**	0.093	0.658	0.133	0.080	0.463	0.487	0.387	0.597	0.996	0.189	0.460	0.543

[1] Country abbreviations, UK=United Kingdom, USA=United States of America

[2] Significant *p*-values (≤ 0.05) are highlighted in bold.
